# Development of an Emergency Department Safety Checklist through a global consensus process

**DOI:** 10.1007/s11739-024-03760-y

**Published:** 2024-10-01

**Authors:** Lucrezia Rovati, Daniele Privitera, Alexander S. Finch, John M. Litell, Autumn M. Brogan, Aysun Tekin, Claudia Castillo Zambrano, Yue Dong, Ognjen Gajic, Bo E. Madsen, for the CERTAIN ED Study Group, Hong Hieu Truong, Nasrin Nikravangolsefid, Mahmut Can Ozkan, Amos Lal, Oguz Kilickaya, Alexander S. Niven, Emily Aaronson, Derar H. Abdel-Qader, Divya E. Abraham, Pablo Aguilera, Saima Ali, Maryam Bahreini, Anish Baniya, Fernanda Bellolio, Jochen Bergs, Hjalti Mar Bjornsson, Alberto Bonfanti, Jesica Bravo, Caitlin S. Brown, Basil Bwambale, Nicolò Capsoni, Enrique Casalino, Lucas B. Chartier, Sandeep N. David, Sagun Dawadi, Mirko Di Capua, Melis Efeoglu, Leila Eidinejad, Doris Eis, Ulf Ekelund, Cenker Eken, Yonathan Freund, Brian Gilbert, Davide Giustivi, Shamai Grossman, Saïd Hachimi Idrissi, Kim Hansen, Chorng-Kuang How, Katrin Hruska, Aamir Ghafoor Khan, Henrik Laugesen, Lars Erik Laugsand, Lawrence Kule, Le Thi Thanh Huong, Mate Lerga, Marta Macias Maroto, Nataša Mavrinac, Walter Menacho Antelo, Nalan M. Aksu, Tatjana Mileta, Talayeh Mirkarimi, Victor Mkanyu, Neema Mnape, Afif Mufarrij, Muhammed Elhady Muhammed Elgasim, Visnja Nesek Adam, Tran Ngoc Thuy Hang, Nguyen Xuan Ninh, Seyedeh Zahra Nouri, Kei Ouchi, Sowjanya Patibandla, Pham Tien Ngoc, Ingrid Prkačin, Emma Redfern, Alejandro-Antonio Rendón Morales, Roberta Scaglioni, Lindy Scholten, Belinda Scott, Nima Shahryarpour, Optatus Silanda, Lucas Silva, Tiong Beng Sim, Ksenija Slankamenac, Jonathan Sonis, Maša Sorić, Yuqiang Sun, Nguyen Thai Tri, Tran Viet Quoc, Salim Kemal Tunceri, Joseph Turner, Marie C. Vrablik, Mohamed Wali, Xiaoxv Yin, Sana Zafar, Abedi S. Zakayo, Jian-cang Zhou, Didi Delalic, Sveva Anchise, Marta Colombo, Marco Bettina, Laura Ciceri, Fausto Fazzini, Rossella Guerrieri, Valeria Tombini, Annalisa Geraneo, Annamaria Mazzone, Cristina Alario, Elisa Bologna, Eugenia Rocca, Giorgio Parravicini, Greta Li Veli, Ilaria Paduanella, Marianna Sanfilippo, Matteo Coppola, Michela Rossini, Simone Saronni

**Affiliations:** 1 Department of Medicine and Surgery, University of Milano-Bicocca, Milan, Italy; 2 Mayo Clinic, Department of Medicine, Division of Pulmonary and Critical Care Medicine, 200 First Street SW, Rochester, 55905 USA; 3 ASST Grande Ospedale Metropolitano Niguarda, Department of Emergency Medicine, Milan, Italy; 4 Department of Emergency Medicine, Mayo Clinic, Rochester, MN USA; 5 Department of Emergency Medicine, Hennepin Healthcare, Minneapolis, MN USA; 6 Department of Anesthesiology and Perioperative Medicine, Mayo Clinic, Rochester, MN USA

**Keywords:** Emergency department, Patient safety, Medical errors, Checklist, Quality of health care, Delphi technique

## Abstract

Emergency departments (EDs) are at high risk for medical errors. Checklist implementation programs have been associated with improved patient outcomes in other high-risk clinical settings and when used to address specific aspects of ED care. The aim of this study was to develop an ED Safety Checklist with broad applicability across different international ED settings. A three-round modified Delphi consensus process was conducted with a multidisciplinary and multinational panel of experts in emergency medicine and patient safety. Initial checklist items were identified through a systematic review of the literature. Each item was evaluated for inclusion in the final checklist during two rounds of web-based surveys and an online consensus meeting. Agreement for inclusion was defined a priori with a threshold of 80% combined agreement. Eighty panel members from 34 countries across all seven world regions participated in the study, with comparable representation from low- and middle-income and high-income countries. The final checklist contains 86 items divided into: (1) a general ED Safety Checklist focused on diagnostic evaluation, patient reassessment, and disposition and (2) five domain-specific ED Safety Checklists focused on handoff, invasive procedures, triage, treatment prescription, and treatment administration. The checklist includes key clinical tasks to prevent medical errors, as well as items to improve communication among ED team members and with patients and their families. This novel ED Safety Checklist defines the essential elements of high-quality ED care and has the potential to ensure their consistent implementation worldwide.

**Supplementary Information:**

The online version contains supplementary material available at 10.1007/s11739-024-03760-y.

## Introduction

The emergency department (ED) plays a crucial role in healthcare delivery, serving as the first point of contact for patients in critical moments of need. While it can provide rapid access to healthcare resources for high-acuity patients, the ED is a setting at significant risk for medical errors that threaten patient safety [1]. Accurately estimating the rate of medical errors in the ED is challenging due to underreporting and a lack of standardized metrics [2, 3]. Measurable outcomes of ED errors include preventable patient morbidity and mortality, increased hospitalizations and ED return visits, delays in care, and an overall poor patient experience [4]. International organizations have called attention to the importance of improving quality and safety in the ED, but effective systems-based solutions are still lacking [5, 6].

Checklist implementation programs have been associated with improved patient outcomes in other high-risk clinical settings, including the intensive care unit (ICU) and the operating room [7–10]. In emergency medicine, checklists have been developed to reduce errors during invasive procedures, handoffs, and the management of specific clinical scenarios [11–19]. However, a universal safety framework for patients presenting to the ED has not yet been implemented in clinical practice.

To define the components of high-quality ED care that should be consistently implemented to reduce medical errors, we conducted a multinational and multidisciplinary Delphi study with experts in emergency medicine and patient safety. The aim of this study was to develop an ED Safety Checklist with broad applicability in different international ED settings.

## Methods

### Study design

We conducted a three-round modified Delphi consensus process between April 2023 and July 2023 [20]. The study consisted of two rounds of web-based surveys followed by an online consensus meeting [21, 22]. The project was based on the Checklist for Early Recognition and Treatment of Acute Illness and iNjury (CERTAIN) program, a global ICU quality improvement initiative that provides evidence-based checklists for structured ICU admission and rounding [8], and was named CERTAIN ED. The study protocol was reviewed and approved as exempt by the Mayo Clinic Institutional Review Board. Informed consent to participate in the study was obtained from each panelist prior to the initiation of Round 1. The study is reported following the Standards for Reporting Qualitative Research (SRQR) Reporting Guidelines.

### Selection of participants

The core study group was composed of physicians and nurses with previous clinical and research experience in emergency medicine and critical care medicine. Panel members were recruited using an iterative sampling approach that combined purposive and snowball sampling [22]. Experts in emergency medicine and patient safety were identified by the core study group and invited to participate in the Delphi consensus. Eligibility requirements included practicing in the general ED and having a special interest in patient safety. Potential panelists included peer review and quality assurance committee members identified through the researchers' professional network and the CERTAIN network, authors of peer-reviewed publications in the field of ED quality and safety, and members of emergency medicine professional organizations actively involved in patient safety and quality improvement initiatives. Invited panel members were encouraged to suggest additional experts from their professional networks. All potential panelists were screened before distributing the Round 1 survey link and selected primarily based on their publication record or involvement in ED quality and safety initiatives. In addition, a deliberate effort was made to ensure multidisciplinary and geographically diverse representation on the panel. All participants who completed Round 1 were invited to participate in Rounds 2 and 3. No new experts were recruited after the completion of Round 1. All experts were given the opportunity to be identified as collaborative authors of the study.

### Systematic review

The core study group conducted a review of the published literature on medical errors that occur during the care of patients in the general ED. Studies that focused on pediatric or other specialty emergency care were excluded, as were studies that evaluated errors occurring during specific procedures or related only to specific conditions. Search strategies were developed by a medical librarian using a combination of keywords and standardized index terms and five databases (Cochrane Central Register of Controlled Trials, Embase, Medline, CINAHL, and Web of Science) were searched from inception through November 2022, with no language restrictions. Full search strategies are provided in the Supplementary Appendix 1. Two authors independently screened publications for inclusion, and discrepancies were resolved by discussion among the review team. The study selection process was conducted and managed using Covidence. After evaluation of the abstracts and full-text manuscripts retrieved by the search, 116 studies were included in the final analysis (Supplementary Fig. 1 and Supplementary Bibliography).

### Data collection and checklist development

Empirical evidence from a total of 116 studies, along with tacit knowledge from the study group members clinical experience, was used to develop the initial checklist items. Potential checklist items consisted of clinical and communication tasks that, when omitted, are associated with the occurrence of medical errors in the ED. Each item was evaluated for inclusion in the final checklist during two rounds of web-based surveys. Surveys were developed and distributed using Research Electronic Data Capture version 8.11.11 (REDCap, Vanderbilt University, Nashville, Tennessee, USA) for Round 1 and Qualtrics XM Platform (Qualtrics, Provo, UT, USA) for Round 2. In both surveys, experts were able to provide free-text comments to suggest changes to individual checklist items or to propose new items.

During Round 1 (April 12–May 22, 2023), each proposed item was rated for inclusion in the checklist by Delphi panel members using a five-point Likert scale (strongly disagree, disagree, undecided, agree, strongly agree). Consensus for inclusion was defined a priori using a threshold of 80% of combined agreement (agree or strongly agree) [23, 24]. Items for which consensus for inclusion was not reached were either discarded or modified based on the results of qualitative analysis of free-text comments and after discussion among the core study group. Participant information and hospital characteristics were also collected in Round 1. Finally, participants were asked which checklists they used in their routine ED practice and how they would like to access the new checklist.

Checklist items that did not reach consensus for inclusion in Round 1 were modified and rated by the panelists in Round 2 (May 31–June 26, 2023), along with new items that emerged from qualitative analysis of experts’ comments. A four-point Likert scale (strongly disagree, somewhat disagree, somewhat agree, strongly agree) and a pre-defined 80% threshold for combined agreement (somewhat agree and strongly agree) were used during Round 2. The checklist was further revised by the core study group based on qualitative analysis of free-text comments. Participants were also asked to rate the likelihood of benefit from using each section of the checklist for different professional categories, as well as the overall usefulness of each section, using a 5-point Likert scale.

An online consensus meeting (Round 3) of the panel and core study group members was held on July 13, 2023 via Zoom videoconferencing platform (Zoom Video Communications Inc, San Jose, CA, USA) to finalize the checklist and discuss potential implementation strategies. The meeting was recorded and shared with the Delphi panel members as an unlisted video on YouTube (Google LLC, San Bruno, CA, USA) to allow asynchronous viewing. YouTube analytics were used to determine the number of unique viewers of the recording prior to July 31, 2023. The final checklist was also shared with all panel members for approval, and participants were given the opportunity to provide additional comments via email over the next 2 weeks.

### Data analysis

The denominator used to calculate the level of combined agreement was the total number of participants who completed each round. Descriptive statistics were reported as numbers and percentages. Differences in combined agreement by income level for country of practice (high-income countries, HICs vs. low- and middle-income countries, LMICs) were assessed using Fisher exact test. A P value < 0.05 was considered statistically significant. Stata Statistical Software version 17 (StataCorp LLC, College Station, TX, USA) was used to perform the analyses.

Collected free-text comments were reviewed individually by two members of the core study group (L.R. and A.T. or C.C.Z.) and organized into themes. After each round, the results of the thematic analysis were discussed by the core study group and used to revise the checklist.

## Results

**Fig. 1:**
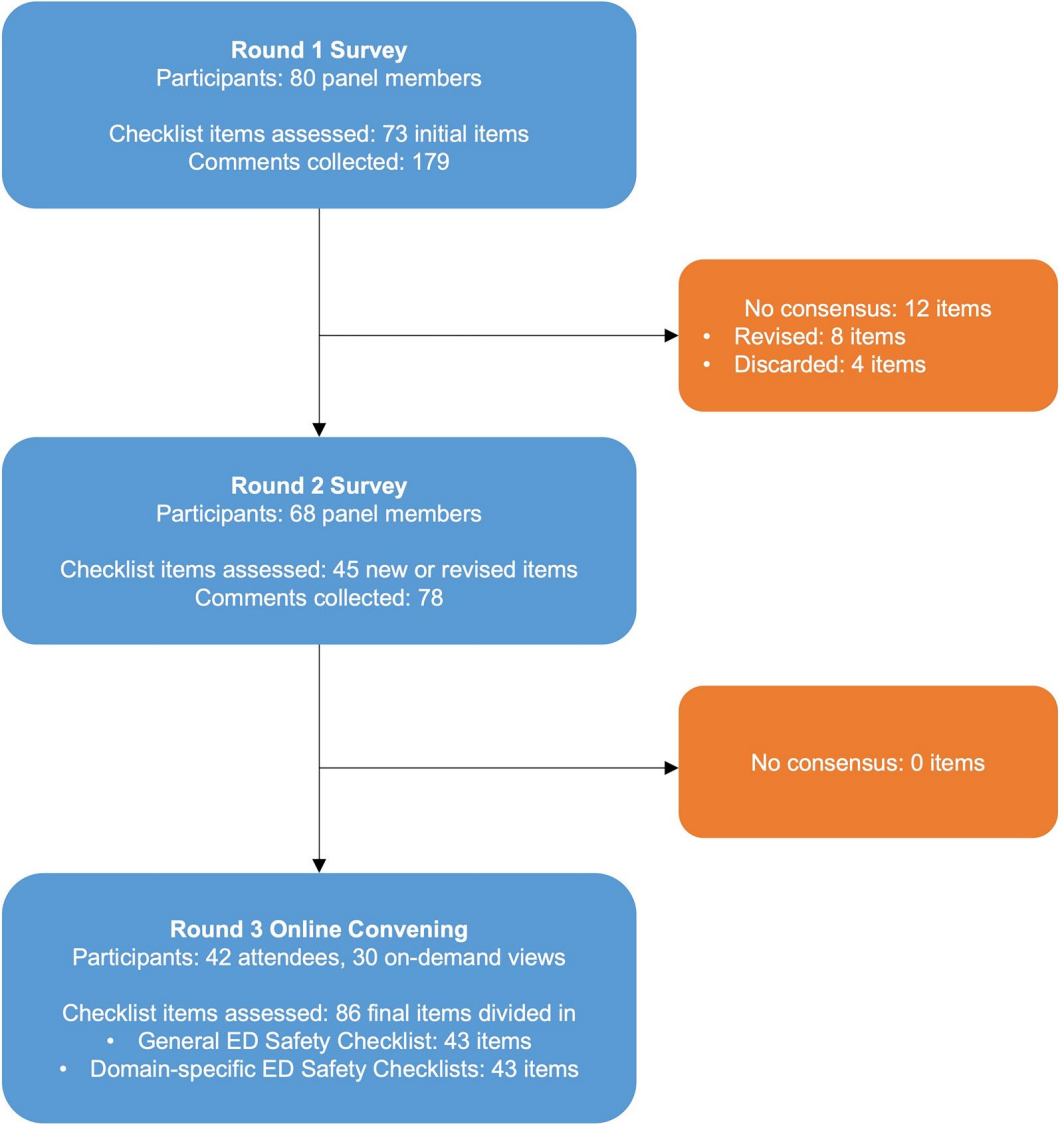
Consensus development process flowchart. Summary of the modified three-round Delphi process. Initial checklist items were identified by the core study team through a systematic review of the literature on medical errors in the emergency department

The three-round Delphi process is summarized in Fig. 1 and described in detail in the Supplement.

### Delphi panel characteristics

Of the 157 invited participants, 80 emergency medicine and patient safety experts completed Round 1, while 5 surveys were returned incomplete (51% response rate, 94% completion rate). The characteristics of the 80 panel members are summarized in Table 1. The panel consisted of clinicians actively practicing emergency medicine in 56 cities of 34 countries across all seven world regions, with comparable representation from LMICs (37/80, 46%) and HICs (43/80, 54%) according to the World Bank classification [25]. Most of the panelists were emergency medicine physicians (56/80, 65%), but physicians from other specialties, as well as nurses, pharmacists, and a hospital administrator also participated in the study. Among participants, 58% (46/80) had more than 10 years of ED experience and 85% (68/80) practiced in academic medical centers.

**Table 1:** Expert panel characteristics

Characteristic (*n* = 80)	Delphi panel members no. (%)
Gender	
Female	32 (40)
Male	48 (60)
Profession	
Physician	68 (85)
Nurse	7 (9)
Pharmacist	4 (5)
Health administrator	1 (1)
Specialty (*n* = 68)	
Emergency medicine	56 (82)
Internal medicine	3 (4)
No specialty	6 (9)
Other	3 (4)
Years of ED experience	
0–5	14 (18)
6–10	20 (25)
11–15	18 (23)
16–20	13 (16)
Over 20	15 (19)
Global region^a^	
East Asia and Pacific	15 (19)
Europe and Central Asia	30 (38)
Latin America and the Caribbean	5 (6)
Middle East and North Africa	6 (8)
North America	10 (13)
South Asia	7 (9)
Sub-Saharan Africa	7 (9)
Country income level^a^	
Low income	5 (6)
Lower middle income	24 (30)
Upper middle income	8 (10)
High income	43 (54)
Hospital setting	
Academic medical center	68 (85)
Non-academic medical center	12 (15)

^a^Country income level and global region correspond to World Bank classification for 2023

### The ED Safety Checklist

**Fig. 2:**
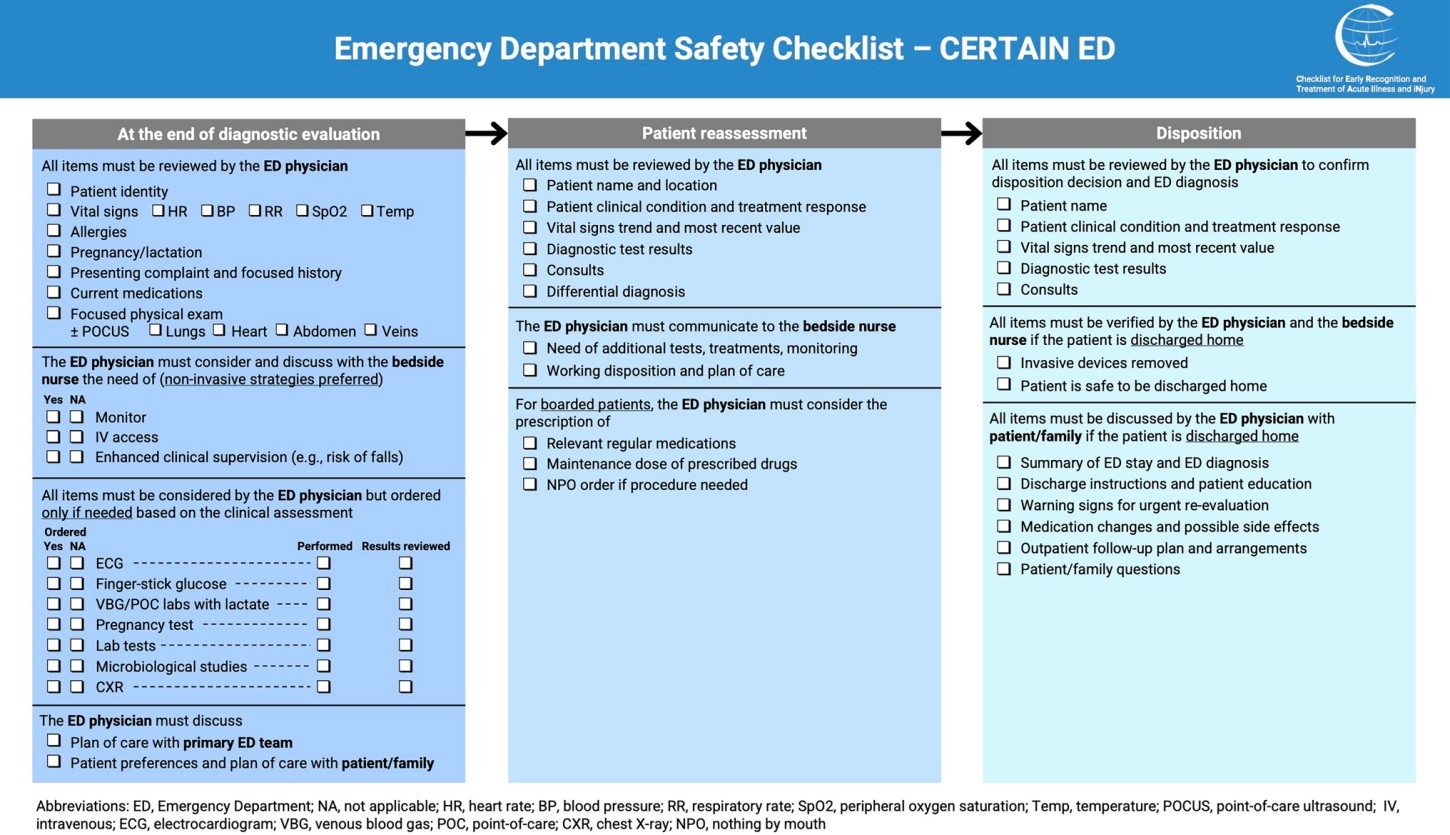
General Emergency Department Safety Checklist

**Fig. 3:**
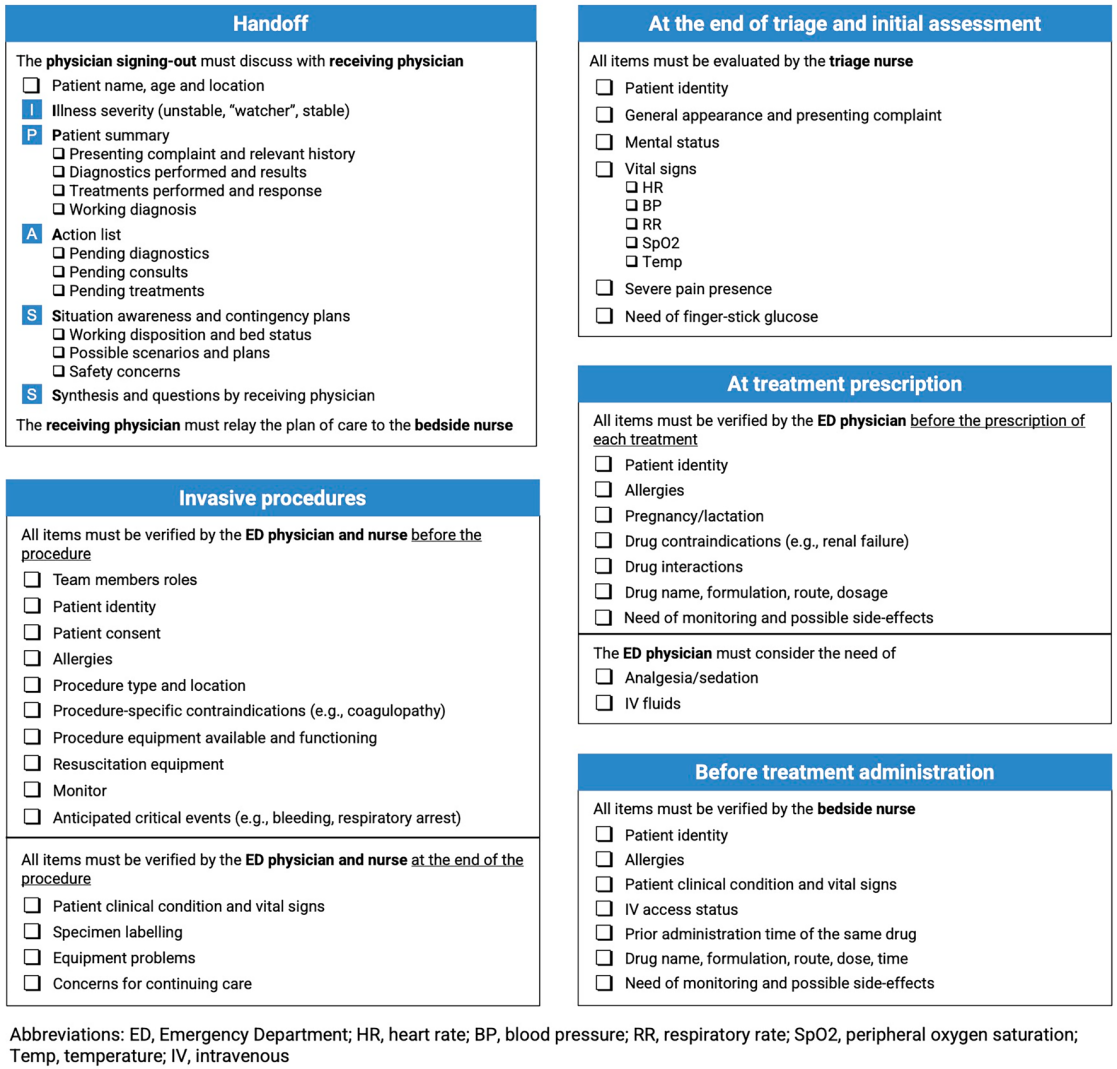
Domain-specific Emergency Department Safety Checklists

**Fig. 4:**
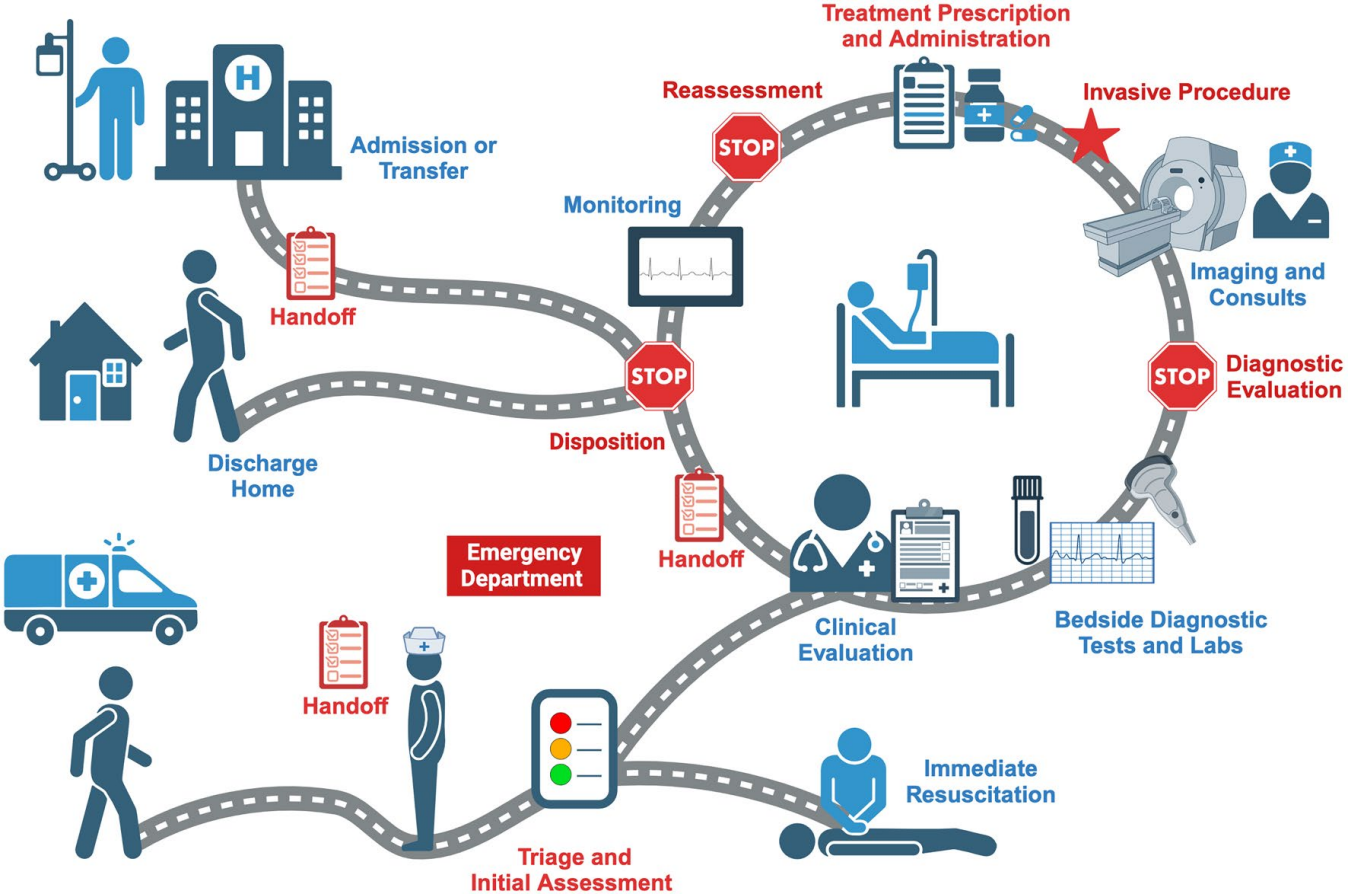
Patient flow through the emergency department. Infographic describing the patient flow from arrival in the Emergency Department (ED) through discharge or admission to the hospital. The general ED Safety Checklist is designed for use at three critical junctures (indicated by the stop signal) where the clinician can pause to verify that all important tasks have been completed. The five domain-specific Safety Checklists complement the general checklist by aiding clinicians during other key moments in the ED care process (colored in red)

The checklist developed through the Delphi process contained 86 items, organized into a general ED Safety Checklist (Fig. 2) and five domain-specific Safety Checklists (Fig. 3). The general ED Safety Checklist focused on three critical timepoints in the patient flow through the ED: initial diagnostic evaluation, patient reassessment, and disposition (Fig. 4). The five domain-specific Safety Checklists focused on handoff, invasive procedures, triage and initial assessment, treatment prescription, and treatment administration.

Three types of items were included in the checklist. First, important clinical actions that should be performed for all patients presenting to the ED, such as assessing for allergies or checking vital signs. Second, diagnostic tests and treatments that are considered in the care of most ED patients but are performed only in specific cases according to clinical judgment and perceived patient need. Examples include obtaining an electrocardiogram (ECG) or administering appropriate analgesia. Finally, prompts to improve communication among ED team members and with patients and their families were included in the checklist.

Checklist items that achieved the highest levels of agreement were related to information sharing at handoff and discharge, to clinical assessment, with particular emphasis on vital signs review, and to prevention of so-called never events such as iatrogenic allergic reactions, wrong patient errors, and equipment failure during invasive procedures (Supplementary Tables 1 and 2). Items discarded from the checklist were largely clerical duties, such as writing clinical notes, and invasive diagnostic tests and procedures, such as urinary catheter placement.

### Differences in agreement by country income level

The results of the first two rounds of the Delphi process were stratified by the income level of the country of practice of the panel members. Checklist items that did not reach consensus for inclusion in only one of the two groups are shown in Supplementary Table 3. In general, the proportion of participants agreeing to include specific checklist items was lower in HICs compared to LMICs.

### Checklist implementation strategies

At the beginning of the Delphi process, 25% (20/80) of panelists reported not using any checklist in their daily ED practice. Only a minority of participants reported using a general ED Safety Checklist (22/80, 28%) or a handoff checklist (23/80, 29%), while procedure-specific checklists (39/80, 49%) and syndrome- or disease-specific protocols (32/80, 40%) were more commonly used.

The majority of panel members (54/80, 68%) suggested implementing the new checklist as an electronic tool embedded in the electronic health record (EHR). Other implementation options included displaying the checklist as a wall poster in different areas of the ED (30/80, 38%), providing it to clinicians as a standalone mobile or web application (28/80, 35%), as a printout for each patient (19/80, 24%), or as a personal pocketbook (12/80, 15%).

Resident physicians were identified as the professional category most likely to benefit from the newly developed checklist, except for the triage and treatment administration sections, for which nurses were recognized as the primary users (Supplementary Fig. 2). Panel members also reported that attending physicians could benefit from the proposed checklist. At least 75% of respondents found each section of the checklist either very useful or extremely useful (Table 2). The two sections considered most useful were the handoff and procedure sections.

**Table 2:** Rating of the eight checklist sections by the Delphi panel members

Checklist section	Not at all useful no. (%)	Slightly useful no. (%)	Moderately useful no. (%)	Very useful no. (%)	Extremely useful no. (%)
Diagnostic evaluation (*n* = 67)	1 (2)	4 (6)	10 (15)	22 (33)	30 (45)
Patient reassessment (*n* = 67)	0 (0)	5 (8)	8 (12)	27 (40)	27 (40)
Disposition (*n* = 66)	0 (0)	3 (5)	10 (15)	34 (52)	19 (29)
Handoff (*n* = 67)	0 (0)	3 (5)	8 (12)	27 (40)	29 (43)
Invasive procedures (*n* = 68)	1 (2)	1 (2)	8 (12)	27 (40)	31 (46)
Triage (*n* = 67)	2 (3)	2 (3)	13 (19)	23 (34)	27 (40)
Treatment prescription (*n* = 67)	1 (2)	2 (3)	11 (16)	25 (37)	28 (42)
Treatment administration (*n* = 68)	1 (2)	3 (4)	9 (13)	26 (38)	29 (43)

## Discussion

In this study, we report the structured development of an ED Safety Checklist by a global consensus of experts in emergency medicine and patient safety. The checklist constitutes a universal safety framework for patients admitted to the ED and consists of 86 checklist items representing the essential components of high-quality emergency care as identified by the Delphi panel. The checklist was designed to be broadly applicable in both HICs and LMICs, and the majority of panelists indicated that the checklist would be very useful.

The unique operational characteristics of the ED, including rapid patient turnover, interruptions, and overcrowding, can cause healthcare providers to inadvertently overlook critical steps in patient care, resulting in preventable adverse events and delays in care. Several studies have shown that most medical errors in the ED occur when routine and seemingly simple tasks, such as checking vital signs or ordering an ECG, are missed [26]. Checklists can reduce omissions by explicitly outlining the minimum steps required in a process and ensuring their consistent execution, even under stressful conditions [27]. Although several checklists have been developed for use in the ED, including procedural and handoff checklists, as well as disease-specific diagnostic and treatment algorithms [11–18, 28–33], up to 25% of panelists reported not using a checklist in routine clinical practice. In addition, only two checklists addressing the general ED care process have been described in the literature. These include an ED Safety Checklist developed in the United Kingdom to facilitate regular monitoring of patients by nurses, and a Medical Emergency Checklist designed by the World Health Organization (WHO) as a modification of the WHO Trauma Care Checklist for the initial assessment and resuscitation of critically ill patients [19, 34–36]. The novel ED Safety Checklist described in this study incorporates items from both these checklists and other previously validated tools, such as the IPASS handoff mnemonic [37], to provide a comprehensive, evidence-based framework that can be consistently implemented in clinical practice. In addition, to facilitate rapid verification that all essential tasks have been completed at the appropriate time, the items are organized into different checklist sections that can be quickly reviewed at critical junctures in the patient flow through the ED.

During the consensus process, panel members agreed that the checklist should focus on essential actions for the clinical assessment of the patient rather than on clerical duties. In addition, the use of history and physical examination findings, along with inexpensive and readily available bedside diagnostic tools, was favored over the use of more advanced diagnostic tools [38]. This was considered particularly important by participants practicing in LMICs, where point-of-care laboratory tests and imaging modalities are frequently the only available option [39]. This factor and the lack of other medical error prevention strategies, such as electronic prescribing systems, may have contributed to the higher levels of agreement observed among participants from LMICs. It is important to emphasize that the checklist does not promote the systematic performance of all proposed diagnostic tests in every patient, but rather encourages their consideration and selection based on clinical judgment. By prompting the clinician to systematically stop and think about what tests and treatments are needed for a patient, the checklist has the potential to reduce the routine performance of some procedures, such as IV placement, that may not always be necessary. In addition to addressing important clinical tasks, the checklist also includes elements to improve teamwork and shared decision-making [40]. Interestingly, the items with the highest level of agreement were those related to information exchange at handoff and discharge. Communication failures have already been identified as a major risk factor for medical errors in the ED [41, 42]. Moreover, other checklists, such as the WHO Surgical Safety Checklist, have been proposed to reduce adverse events by improving teamwork and communication [9, 43].

Implementation strategies suggested by participants varied, with checklist integration into the EHR with the ability to highlight only missing tasks being the preferred alternative. However, other strategies that may be more feasible in LMICs, such as displaying the checklist as a wall poster that can either be read aloud or completed on a dry-erase board, were also well received. Checklist content, display strategy, and user assignment should be tailored to the unique workflow of each ED and the specific roles and responsibilities of clinicians [40]. Deciding who reviews the checklist can be an opportunity to empower team members who are less likely to speak up, such as residents and nurses, by allowing them to provide input and feedback in high-risk situations. Importantly, the checklist does not need to be an additional administrative document to be filled out, adding to the clinician's workload, but can serve as a guide to assist providers in moments of need, especially if they are less experienced or practicing in countries with less developed emergency care systems. In this sense, a digital checklist that fills in automatically as information is added to the EHR may be the best solution, where feasible, to keep track of what has been done without increasing paperwork for practicing clinicians. Where paper medical charts are still in use, structuring them according to the items on the checklist may be one way to promote systematic patient assessment. Finally, the checklist can also be used as a teaching tool to aid residents prioritize important emergency care tasks, or as an assessment tool to evaluate the quality of care provided in different international EDs.

This study has several limitations. First, the Delphi panel was composed mainly of physicians, with limited representation of nurses and allied health professionals and no patient representation. This is partly explained by the strategy used to recruit panel members, which relied largely on publication records and may therefore have introduced a selection bias towards physicians. As Delphi studies use a non-random sampling technique to ensure measurable “expertise” of participants in a particular area, there is always an inherent bias in the recruitment process. In the next phases of the project, all stakeholders and not only selected clinicians should be actively involved in further developing the checklist. Second, the effectiveness of the checklist in improving patient outcomes has yet to be evaluated. After its validation and testing, we plan to integrate the checklist into a broader education and quality improvement program that will be implemented in different international ED settings, particularly where emergency care systems are less mature, potentially resulting in greater impact. The checklist will be customized and tailored by individual institutions to align with the specific needs of their ED providers, allowing for modifications based on testing and validation experiences, and a prospective quality improvement trial will be designed to evaluate patient outcomes following the implementation of the CERTAIN ED program in various international EDs. The length of the checklist was another concern highlighted during the Delphi process, since it can lead to "checklist fatigue" [44]. The time required to review each section should be carefully evaluated in a simulated clinical environment to optimize the process [40]. If the checklist proves effective in promoting consistent performance of essential clinical tasks, thereby preventing medical errors while reducing potentially unnecessary tests, its use could lead to an overall reduction in ED length of stay.

## Conclusions

A multidisciplinary and multinational panel of emergency medicine and patient safety experts reached consensus on the essential components of high-quality ED care, ensuring a diverse range of perspectives and experiences. The results of the Delphi process informed the development of a general ED Safety Checklist for use at three critical timepoints in ED care (i.e., end of diagnostic evaluation, patient reassessment, and disposition) and five domain-specific Safety Checklists that can support other key ED actions (i.e., handoff, performance of invasive procedures, triage and initial assessment, treatment prescription and administration). By focusing on key elements of care, the checklist has the potential to increase adherence to best care practices and improve patient-centered outcomes in different international ED settings.

## Supplementary Information

Below is the link to the electronic supplementary material.

Supplementary file1 (DOCX 121 KB)

Supplementary file2 (DOCX 22KB)

## Data Availability

The datasets generated and analyzed during the current study are available from the corresponding author on reasonable request.
