# Supplementary material for: Development of an Emergency Department Safety Checklist through a global consensus process
Source: Intern Emerg Med. 2024 Oct 1;20(4):1203–13. doi: 10.1007/s11739-024-03760-y (PMC12130094; doi:10.1007/s11739-024-03760-y)
Supplement: Supplementary file 1 — Supplementary file1 (DOCX 121 KB) [file 11739_2024_3760_MOESM1_ESM.docx]

**Supplementary Information**

**Journal: Internal and Emergency Medicine**

**Title: Development of an Emergency Department Safety Checklist through a Global Consensus Process**

**Authors:** Lucrezia Rovati, MD; Daniele Privitera, CCRN; Alexander S. Finch, MD; John M. Litell, DO; Autumn M. Brogan, MD, MPH; Aysun Tekin, MD; Claudia Castillo Zambrano, MD; Yue Dong, MD; Ognjen Gajic, MD; Bo E. Madsen, MD, MPH; for the CERTAIN ED Study Group

**Corresponding Author:**

Lucrezia Rovati, MD

Department of Medicine, Division of Pulmonary and Critical Care Medicine

Mayo Clinic

200 First Street SW

Rochester, MN 55905, USA

rovati.lucrezia@mayo.edu

**Index:**

- Supplementary Results. Detailed description of the Delphi process
- Supplementary Table 1. Round 1 results
- Supplementary Table 2. Round 2 results
- Supplementary Table 3. Differences in combined agreement according to the income level of the panel members’ country of practice
- Supplementary Figure 1. PRISMA flow diagram for the identification of studies for the systematic review
- Supplementary Figure 2. User groups most likely to benefit from the new checklist
- Supplementary Bibliography
- Supplementary Appendix 1. Search strategy for the systematic review
- Supplementary Appendix 2. Free-text comments collected during Round 1 and 2 of the Delphi process

**Supplementary Results. Detailed description of the Delphi process**

**Round 1**

Seventy-three potential checklist items developed after reviewing the literature (**Supplementary Figure 1 and** **Supplementary Bibliography**) on Emergency Department (ED) medical errors were evaluated in Round 1. The items were organized into 8 checklist sections: triage, diagnostic evaluation, treatment prescription, treatment administration, invasive procedures, patient reassessment, handoff, and discharge. The quantitative results of agreement and disagreement for the checklist items of Round 1 are summarized in **Supplementary Table 1**.

Twelve items did not reach consensus for inclusion in the checklist: completing all ED notes, ordering all tests and consults, assessing the patient's weight, discussing the plan of care with the patient/family and with the ED care team, discussing goals of care and code status with the patient/family, and assessing the need for POCUS, Foley catheter placement, ABO blood type, point of care labs, blood/urine cultures, and chest x-ray. Eight of these items were rephrased and voted again in Round 2, while the remaining four items (completing all ED notes, ordering all tests and consults, assessing the need Foley catheter placement and ABO blood typing) were discarded from the next versions of the checklist.

Forty-three items achieved greater than 90% combined agreement, with two items (discussion of discharge instructions with the patient/family and discussion of follow-up plan with the patient/family) achieving 100% combined agreement. Assessment of vital signs, allergies, and patient identity were the items with the highest number of "strongly agree" responses.

A total of 179 comments were collected and analyzed in Round 1. Comments were used to modify the proposed checklist items and to develop new items that were evaluated in Round 2.

**Round 2**

Of the 80 panel members, 68 completed the second Delphi survey (response rate 85%), with 35 (51%) participants practicing in HICs. In Round 2, participants evaluated 45 new or modified checklist items that were developed based on comments collected and analyzed in the Round 1. New items mainly focused on patient clinical assessment and improved communication within the ED team and with patients. The quantitative results of agreement and disagreement for the checklist items evaluated in Round 2 are summarized in **Supplementary Table 2**.

Consensus for inclusion in the checklist was reached on all 45 items, with 36 items achieving greater than 90% combined agreement. Items that achieved the highest levels of agreement were items related to improving information sharing at handoff and discharge, and items related to patient reassessment and evaluation of treatment response. Verifying the availability and function of equipment before performing an invasive procedure was also considered a key clinical action by 99% of respondents.

Seventy-eight comments were collected and analyzed in Round 2 and used to create the version of the checklist that was finalized in Round 3.

**Round 3**

A total of 42 participants from 12 countries attended the online meeting, with 17 participants from the core study team and 25 participants from the Delphi panel. The recording of the meeting was made available to all Delphi panel members and had 30 viewers over the following 2 weeks. Quantitative and qualitative data collected and analyzed during the first two rounds were used to create the version of the checklist that was evaluated during Round 3. The final checklist evaluated in Round 3 contained a total of 86 items, organized into a general ED Safety Checklist (**Figure 2**) and five domain-specific Safety Checklists (**Figure 3**). The Delphi panel members approved the final checklist, and no further changes were suggested after the online meeting.

**Supplementary Table 1. Round 1 results**

| **Checklist item** | **Combined agreement^a^** | **Combined disagreement^b^** | **Undecided^c^** |
| --- | --- | --- | --- |
| **Triage** |  |  |  |
| Check patient identity | 91% | 4% | 5% |
| Assess vital signs (HR, BP, RR, SpO2) | 99% | 0% | 1% |
| Assess mental status | 98% | 0% | 3% |
| Assess presence and level of pain | 93% | 1% | 6% |
| **Diagnostic evaluation** |  |  |  |
| Check patient identity | 94% | 4% | 3% |
| Review vital signs (HR, BP, RR, SpO2) | 96% | 1% | 3% |
| Review patient temperature | 93% | 3% | 5% |
| Assess mental status | 95% | 0% | 5% |
| Assess presence and level of pain | 95% | 1% | 4% |
| Assess allergies | 90% | 3% | 8% |
| Discuss the goals of care and code status | 74% | 8% | 19% |
| Consider if a monitor is needed | 88% | 5% | 8% |
| Consider if and which type of IV access is needed | 86% | 4% | 10% |
| Consider if urinary catheter is needed | 70% | 18% | 13% |
| Consider if point of care ultrasound is needed | 70% | 13% | 18% |
| Consider if pregnancy test is needed | 86% | 4% | 10% |
| Consider if glucose finger-stick test is needed | 88% | 1% | 11% |
| Consider if point of care labs/arterial blood gas are needed | 78% | 8% | 15% |
| Consider if ECG is needed | 93% | 3% | 5% |
| Consider if chest x-ray is needed | 80% | 4% | 16% |
| Consider if blood/urinary cultures are needed | 76% | 10% | 14% |
| Consider if ABO blood typing is needed | 71% | 10% | 19% |
| Place all tests/consults orders | 73% | 10% | 18% |
| Complete all notes | 66% | 20% | 14% |
| Discuss the plan of care with the ED care team | 79% | 8% | 14% |
| Discuss the plan of care with the patient/family | 76% | 5% | 19% |
| **Treatment prescription** |  |  |  |
| Check patient identity | 95% | 1% | 4% |
| Check patient weight | 73% | 13% | 15% |
| Check patient allergies | 95% | 3% | 3% |
| Check if the patient is pregnant/lactating | 91% | 1% | 8% |
| Check if there are other contraindications to therapy | 89% | 4% | 8% |
| Check if there are any drug interactions | 85% | 1% | 14% |
| Check prescribed drug name. formulation. route of administration | 93% | 1% | 6% |
| Check prescribed dosage | 95% | 1% | 4% |
| Consider if dosage correction for renal function is needed | 86% | 3% | 11% |
| Consider if oxygen/ventilatory support therapy is needed | 86% | 8% | 6% |
| Consider if IV fluids are needed | 89% | 4% | 8% |
| Consider if antibiotic therapy is needed | 88% | 6% | 6% |
| Consider if analgesia/sedation is needed | 91% | 1% | 8% |
| Consider which of the patient regular medications are needed during ED stay | 88% | 4% | 9% |
| Consider if nothing by mouth order is needed | 88% | 4% | 9% |
| **Treatment administration** |  |  |  |
| Check patient identity | 98% | 1% | 1% |
| Check patient allergies | 98% | 1% | 1% |
| Check the prescribed drug name. formulation. route of administration | 96% | 1% | 3% |
| Check the prescribed drug dosage | 95% | 3% | 3% |
| **Checklist item** | **Combined agreement^a^** | **Combined disagreement^b^** | **Undecided^c^** |
| Check if the drug has already been administered | 94% | 3% | 4% |
| Consider if patient monitoring is needed | 91% | 5% | 4% |
| **Invasive procedures** |  |  |  |
| Check patient identity | 96% | 1% | 3% |
| Check contraindications | 94% | 1% | 5% |
| Obtain informed consent | 96% | 1% | 3% |
| Consider if patient monitoring is needed | 95% | 1% | 4% |
| Consider if resuscitation equipment is needed | 96% | 1% | 3% |
| Check site of procedure | 94% | 1% | 5% |
| Check specimens' labels | 94% | 1% | 5% |
| **Patient reassessment** |  |  |  |
| Check patient identity and location | 86% | 6% | 8% |
| Review and interpret all diagnostic test results | 96% | 1% | 3% |
| Review all consultations | 85% | 3% | 13% |
| Review the vital signs trend | 98% | 0% | 3% |
| Assess presence and level of pain | 90% | 0% | 10% |
| Document all critical information | 90% | 3% | 8% |
| **Handoff** |  |  |  |
| Check patient identity and location | 98% | 0% | 3% |
| Discuss the results of the tests/procedures performed | 98% | 0% | 3% |
| Discuss the treatments administered | 95% | 0% | 5% |
| Discuss the plan of care | 96% | 0% | 4% |
| Discuss which results are still pending | 99% | 0% | 1% |
| Discuss which tasks still need to be completed | 96% | 0% | 4% |
| **Discharge** |  |  |  |
| Check patient identity | 90% | 5% | 5% |
| Review and interpret all diagnostic test results | 98% | 1% | 1% |
| Review all consultations | 93% | 3% | 5% |
| Discuss the discharge instructions with the patient/family | 100% | 0% | 0% |
| Discuss the follow-up plan with the patient/family | 100% | 0% | 0% |
| Place all medications and tests orders | 95% | 0% | 5% |
| Complete the discharge note | 91% | 1% | 8% |

^a^Combined agreement: percentage of strongly agree + agree responses. Items colored in red did not reach consensus for inclusion in the checklist (combined agreement ≤ 80%).

^b^Combined disagreement: percentage of disagree + strongly disagree responses.

^c^Undecided: percentage of undecided + empty responses.

All percentages are calculated on the total number of respondents for Round 1 (n = 80).

Abbreviations: HR, heart rate; BP, blood pressure; RR, respiratory rate; SpO2, peripheral oxygen saturation; IV, intravenous; ECG, electrocardiogram; ED, emergency department.

**Supplementary Table 2. Round 2 results**

| **Checklist item** | **Combined agreement^a^** | **Combined disagreement^b^** | **Undecided^c^** |
| --- | --- | --- | --- |
| **Diagnostic evaluation** |  |  |  |
| Review if the patient is pregnant/lactating | 91% | 9% | 0% |
| Review presenting complaint and focused history | 97% | 3% | 0% |
| Review current medications | 97% | 3% | 0% |
| Review focused physical exam findings | 97% | 1% | 1% |
| Consider if point of care US of chest or abdomen or veins is needed | 93% | 7% | 0% |
| Discuss with bedside nurse need of enhanced clinical supervision (e.g., for risk of falls) | 91% | 9% | 0% |
| Consider if arterial blood gas/venous point-of-care labs with lactate are needed | 94% | 6% | 0% |
| Consider if lab tests are needed | 97% | 3% | 0% |
| Consider if microbiological studies are needed | 94% | 4% | 1% |
| Consider if chest x-ray is needed | 96% | 4% | 0% |
| Discuss plan of care with primary ED team | 93% | 6% | 1% |
| Discuss plan of care with patient/family | 93% | 7% | 0% |
| Discuss patient preferences and goals of care | 88% | 10% | 1% |
| **Patient reassessment** |  |  |  |
| Review patient clinical condition and treatment response | 100% | 0% | 0% |
| Review differential diagnosis | 97% | 3% | 0% |
| Communicate to bedside nurse need of additional tests or treatments | 93% | 7% | 0% |
| Communicate to bedside nurse working disposition | 96% | 4% | 0% |
| For boarded patients consider prescription of maintenance dose of prescribed drugs | 87% | 12% | 1% |
| **Handoff** |  |  |  |
| Discuss illness severity (stable, unstable, "watcher") | 100% | 0% | 0% |
| Discuss patient summary (presenting complaint, history, test results, treatment effects, working diagnosis) | 99% | 0% | 1% |
| Discuss situation awareness and contingency plans (working disposition and bed status, possible scenarios and plans, safety concerns) | 97% | 3% | 0% |
| Synthesis by receiving physician and questions | 82% | 16% | 1% |
| Discuss information to relay to bedside nurse | 82% | 18% | 0% |
| **Discharge** |  |  |  |
| Review patient clinical condition | 97% | 3% | 0% |
| Review vital signs trend and most recent values | 96% | 3% | 1% |
| Review differential diagnosis | 81% | 18% | 1% |
| If the patient is discharged home, verify that invasive devices have been removed | 94% | 6% | 0% |
| Verify that the patient is safe to be discharged home | 94% | 4% | 1% |
| Discuss with patient/family the summary of ED stay and diagnosis | 99% | 0% | 1% |
| Discuss with patient/family the home medication changes if the patient is discharged home | 99% | 0% | 1% |
| Discuss with patient/family the warning signs for urgent re- evaluation if the patient is discharged home | 100% | 0% | 0% |
| Discuss the patient/family questions | 96% | 3% | 1% |
| **Triage** |  |  |  |
| Evaluate general appearance and presenting complaint | 97% | 3% | 0% |
| Evaluate temperature | 93% | 7% | 0% |
| Evaluate need of finger-stick glucose | 87% | 10% | 3% |
| **Checklist item** | **Combined agreement^a^** | **Combined disagreement^b^** | **Undecided^c^** |
| **Treatment prescription** |  |  |  |
| Estimate patient weight | 87% | 13% | 0% |
| Prescribe all the relevant symptomatic/supportive therapies | 85% | 15% | 0% |
| Prescribe all the relevant disease-specific therapies | 88% | 12% | 0% |
| **Treatment administration** |  |  |  |
| Vital signs re-assessment considered | 91% | 9% | 0% |
| **Invasive procedures** |  |  |  |
| Verify team members roles | 93% | 7% | 0% |
| Verify allergies | 96% | 4% | 0% |
| Verify procedure equipment available and functioning | 99% | 0% | 1% |
| Discuss anticipated critical events | 97% | 3% | 0% |
| Verify equipment problems at the end of the procedure | 91% | 9% | 0% |
| Discuss concerns for continuing care at the end of the procedure | 91% | 9% | 0% |

^a^Combined agreement: percentage of strongly agree + somewhat agree responses.

^b^Combined disagreement: percentage of somewhat disagree + strongly disagree responses.

^c^Undecided: percentage of empty responses.

All percentages are calculated on the total number of respondents for Round 2 (n = 68).

Abbreviations: US, ultrasound; ED, emergency department.

**Supplementary Table 3. Differences in combined agreement according to the income level of the panel members’ country of practice**

| **Checklist item** | **Low- and middle-income countries**  **No. (%)** | **High-income countries**  **No. (%)** | **P value** |
| --- | --- | --- | --- |
| **Diagnostic evaluation** | **n = 37** | **n = 43** |  |
| Consider if urinary catheter is needed | 30 (81) | 26 (60) | .053 |
| Consider if pregnancy test is needed | 35 (95) | 34 (79) | .055 |
| Consider if glucose finger-stick test is needed | 36 (97) | 34 (79) | .017 |
| Consider if point of care labs/arterial blood gas are needed | 34 (92) | 28 (65) | .006 |
| Consider if chest x-ray is needed | 33 (89) | 31 (72) | .091 |
| Consider if blood/urinary cultures are needed | 31 (84) | 30 (70) | .19 |
| Consider if ABO blood typing is needed | 31 (84) | 26 (60) | .027 |
| Discuss the plan of care with the ED care team | 31 (84) | 32 (74) | .41 |
| Discuss the plan of care with the patient/family | 31 (84) | 30 (70) | .19 |
| **Treatment prescription** | **n = 37** | **n = 43** |  |
| Check patient weight | 31 (84) | 27 (63) | .046 |
| Check if there are any drug interactions | 35 (95) | 33 (77) | .031 |
| Consider if oxygen/ventilatory support therapy is needed | 35 (95) | 34 (79) | .055 |
| Consider which of the patient regular medications are needed during ED stay | 36 (97) | 34 (79) | .017 |
| **Patient reassessment** | **n = 37** | **n = 43** |  |
| Review all consultations | 35 (95) | 33 (77) | .031 |
| **Triage** | **n = 33** | **n = 35** |  |
| Evaluate need of finger-stick glucose | 32 (97) | 27 (77) | .028 |
| **Handoff** | **n = 33** | **n = 35** |  |
| Synthesis by receiving physician and questions | 30 (91) | 26 (74) | .11 |
| Discuss information to relay to bedside nurse | 29 (88) | 27 (77) | .34 |
| **Discharge** | **n = 33** | **n = 35** |  |
| Review differential diagnosis | 31 (94) | 24 (69) | .012 |

**Abbreviations:** ED, emergency department.

**Supplementary Figure 1. PRISMA flow diagram for the identification of studies for the systematic review**

**Identification of studies via databases and registers**

Records removed *before screening*:

Duplicate records removed

(n = 3501)

Records marked as ineligible by automation tools (n = 0)

Records removed for other reasons (n = 0)

Records identified from:

Databases (n = 6928)

- Medline (n = 1824)

- Embase (n = 2000)

- Web of Science (n = 1764)

- CINAHL (n = 1340)

Registers (Central, n = 153)

**Identification**

Records screened

(n = 3580)

Records excluded

(n = 3445)

Reports sought for retrieval

(n = 135)

Reports not retrieved

(n = 0)

**Screening**

Reports assessed for eligibility

(n = 135)

Reports excluded:

Ineligible study design (n = 3)

Ineligible setting (n = 3)

Ineligible topic (n = 13)

Studies included in review

(n = 116)

**Included**

**Supplementary Figure 2. User groups most likely to benefit from the new checklist**

Results of the survey conducted during Round 2 to identify the main users of each checklist section. Participants (n = 68) were asked which ED clinician would benefit from the use of each checklist section at their institution.

**Supplementary Bibliography**

1. Aaronson E, Borczuk P, Benzer T, Mort E, Temin E. 72h returns: A trigger tool for diagnostic error. American journal of emergency medicine 2018;36:359-61.
2. Aaronson E, Jansson P, Wittbold K, Flavin S, Borczuk P. Unscheduled return visits to the emergency department with ICU admission: A trigger tool for diagnostic error. American journal of emergency medicine 2020;38:1584-7.
3. Aaronson EL, Brown D, Benzer T, Natsui S, Mort E. Incident Reporting in Emergency Medicine: A Thematic Analysis of Events. Journal of patient safety 2019;15:E60-E3.
4. Abdel-Qader DH, Al Meslamani AZ, El-Shara AA, et al. Investigating prescribing errors in the emergency department of a large governmental hospital in Jordan. Journal of pharmaceutical health services research 2020;11(4):375-82.
5. Abdel-Qader DH, Saadi Ismael N, Al Meslamani AZ, et al. The Role of Clinical Pharmacy in Preventing Prescribing Errors in the Emergency Department of a Governmental Hospital in Jordan: A Pre-Post Study. Hospital pharmacy 2021;56:681-9.
6. Acheampong F, Tetteh AR, Anto BP. Medication Administration Errors in an Adult Emergency Department of a Tertiary Health Care Facility in Ghana. Journal of patient safety 2016;12:223-8.
7. Adelman JS, Applebaum JR, Schechter CB, et al. Effect of Restriction of the Number of Concurrently Open Records in an Electronic Health Record on Wrong-Patient Order Errors: A Randomized Clinical Trial. Jama 2019;321:1780-7.
8. Adler JL, Gurley K, Rosen CL, Wolfe RE, Grossman SA. Assessing resident and attending error and adverse events in the emergency department. American journal of emergency medicine 2022;54:228-31.
9. Akhil N, Thomas PP, Shivaraj DR, Thomas SM, Londhe SP. Assessment, evaluation, and analysis of the medication errors of the patients admitted at the emergency department of a tertiary care teaching hospital of a South Indian city. Asian Journal of Pharmaceutical and Clinical Research 2017;10(5):161-5.
10. Aksu NM, Akkas M. Where are we in patient safety in the ED in Turkey? Medicine 2019;98:e17569.
11. Alsabri M, Boudi Z, Zoubeidi T, et al. Analysis of Risk Factors for Patient Safety Events Occurring in the Emergency Department. Journal of patient safety 2022;18:e124-e35.
12. Anzan M, Alwhaibi M, Almetwazi M, Alhawassi TM. Prescribing errors and associated factors in discharge prescriptions in the emergency department: A prospective cross-sectional study. PLoS ONE [Electronic Resource] 2021;16:e0245321.
13. Baartmans MC, Hooftman J, Zwaan L, van Schoten SM, Erwich J, Wagner C. What Can We Learn From In-Depth Analysis of Human Errors Resulting in Diagnostic Errors in the Emergency Department: An Analysis of Serious Adverse Event Reports. Journal of patient safety 2022;21:21.
14. Bashkin O, Caspi S, Swissa A, Amedi A, Zornano S, Stalnikowicz R. Human Factors and Quality Improvement in the Emergency Department: Reducing Potential Errors in Blood Collection. Journal of patient safety 2020;16:47-51.
15. Bizovi KE, Beckley BE, McDade MC, et al. The effect of computer-assisted prescription writing on emergency department prescription errors. Academic emergency medicine 2002;9:1168-75.
16. Blank FS, Tobin J, Macomber S, Jaouen M, Dinoia M, Visintainer P. A "back to basics" approach to reduce ED medication errors. Journal of Emergency Nursing 2011;37:141-7.
17. Bonkowski J, Carnes C, Melucci J, et al. Effect of barcode-assisted medication administration on emergency department medication errors. Academic emergency medicine 2013;20:801-6.
18. Brown TW, McCarthy ML, Kelen GD, Levy F. An epidemiologic study of closed emergency department malpractice claims in a national database of physician malpractice insurers. Academic emergency medicine 2010;17:553-60.
19. Cabilan CJ, Hughes JA, Shannon C. The use of a contextual, modal and psychological classification of medication errors in the emergency department: a retrospective descriptive study. Journal of Clinical Nursing 2017;26:4335-43.
20. Canfield C, Udeh C, Blonsky H, Hamilton AC, Fertel BS. Limiting the number of open charts does not impact wrong patient order entry in the emergency department. Journal of the American College of Emergency Physicians open 2020;1:1071-7.
21. Chellis M, Olson J, Augustine J, Hamilton G. Evaluation of missed diagnoses for patients admitted from the emergency department. Academic emergency medicine 2001;8:125-30.
22. Chern CH, Wang LM, How CK. Exploration of clinically significant adverse events in adult non-traumatic emergency department discharged patients through the basic management process analysis - A five-year experience. Journal of Acute Medicine 2012;2(1):19-25.
23. Claret PG, Bobbia X, Renia R, et al. Prescription errors by emergency physicians for inpatients are associated with emergency department length of stay. Therapie 2016;25:25.
24. Clementz A, Jost J, Tchalla A, et al. Implementation and evaluation of validation of prescriptions and pharmaceutical interventions in an adult Emergency Department. Pharmacien Hospitalier et Clinicien 2017;52(2):152-9.
25. Cosby KS, Roberts R, Palivos L, et al. Characteristics of patient care management problems identified in emergency department morbidity and mortality investigations during 15 years. Annals of emergency medicine 2008;51:251-61.
26. Dabaghzadeh F, Rashidian A, Torkamandi H, et al. Medication errors in an emergency department in a large teaching hospital in tehran. Iranian Journal of Pharmaceutical Research 2013;12:937-42.
27. De Andres Lazaro AM, Sevilla Sanchez D, Ortega Romero MD, Codina Jane C, Ribas Sala J, Sanchez Sanchez M. Evaluation of adverse drug reactions in emergency department practice. Emergencias 2013;25:361-7.
28. Dubosh NM, Carney D, Fisher J, Tibbles CD. Implementation of an emergency department sign-out checklist improves transfer of information at shift change. Journal of emergency medicine 2014;47:580-5.
29. Ehsani SR, Cheraghi MA, Nejati A, Salari A, Esmaeilpoor AH, Nejad EM. Medication errors of nurses in the emergency department. Journal of Medical Ethics & History of Medicine 2013;6:11.
30. Fanning L, Jones N, Manias E. Impact of automated dispensing cabinets on medication selection and preparation error rates in an emergency department: a prospective and direct observational before-and-after study. Journal of Evaluation in Clinical Practice 2016;22:156-63.
31. Fordyce J, Blank FS, Pekow P, et al. Errors in a busy emergency department. Annals of emergency medicine 2003;42:324-33.
32. Forster AJ, Rose NG, van Walraven C, Stiell I. Adverse events following an emergency department visit. Quality & Safety in Health Care 2007;16:17-22.
33. Freund Y, Goulet H, Bokobza J, et al. Factors associated with adverse events resulting from medical errors in the emergency department: two work better than one. Journal of emergency medicine 2013;45:157-62.
34. Friedman SM, Provan D, Moore S, Hanneman K. Errors, near misses and adverse events in the emergency department: what can patients tell us? CJEM Canadian Journal of Emergency Medical Care 2008;10:421-7.
35. Gauthier-Wetzel HE. Barcode Medication Administration Software Technology Use in the Emergency Department and Medication Error Rates. CIN: Computers, Informatics, Nursing 2022;40:382-8.
36. George J. Exploring the common prescribing errors that occur in the emergency department. Emergency Nurse 2020;28:17-22.
37. Ghasemi M, Khoshakhlagh AH, Mahmudi S, Fesharaki MG. Identification and assessment of medical errors in the triage area of an educational hospital using the SHERPA technique in Iran. International Journal of Occupational Safety & Ergonomics 2015;21:382-90.
38. Glickman SW, Mehrotra A, Shea CM, et al. A Patient Reported Approach to Identify Medical Errors and Improve Patient Safety in the Emergency Department. Journal of patient safety 2020;16:211-5.
39. Goldstein LN, Morrow LM, Sallie TA, et al. The accuracy of nurse performance of the triage process in a tertiary hospital emergency department in Gauteng Province, South Africa. South African Medical Journal Suid-Afrikaanse Tydskrif Vir Geneeskunde 2017;107:243-7.
40. Gómez SG. Administration Medication Errors in Emergency Department in Level III Hospital. Nure Investigacion 2012:1-9.
41. Goulet H, Guerand V, Bloom B, et al. Unexpected death within 72 hours of emergency department visit: were those deaths preventable? Critical Care (London, England) 2015;19:154.
42. Graber ML, Sorensen AV, Biswas J, et al. Developing checklists to prevent diagnostic error in Emergency Room settings. Diagnosis 2014;1:223-31.
43. Green RA, Hripcsak G, Salmasian H, et al. Intercepting wrong-patient orders in a computerized provider order entry system. Annals of emergency medicine 2015;65:679-86.e1.
44. Gregory H, Cantley M, Calhoun C, Hall GA, Matuskowitz AJ, Weant KA. Incidence of prescription errors in patients discharged from the emergency department. American journal of emergency medicine 2021;46:266-70.
45. Griffey RT, Schneider RM, Sharp BR, et al. Multicenter Test of an Emergency Department Trigger Tool for Detecting Adverse Events. Journal of patient safety 2021;17:e843-e9.
46. Griffey RT, Schneider RM, Todorov AA. Emergency Department Adverse Events Detected Using the Emergency Department Trigger Tool. Annals of emergency medicine 2022;80:528-38.
47. Gurley KL, Burstein JL, Wolfe RE, Grossman SA. Using a rule-based system to define error in the emergency department. Journal of the American College of Emergency Physicians open 2020;1:887-97.
48. Gurley KL, Wolfe RE, Burstein JL, Edlow JA, Hill JF, Grossman SA. Use of Physician Concerns and Patient Complaints as Quality Assurance Markers in Emergency Medicine. The Western Journal of Emergency Medicine 2016;17:749-55.
49. Hajibeglo A, Zagheri Tafreshi M, Kamrani F, Nasiri M. The Impact of Training on Medication Error Rate of the Emergency Department in Hospitals Affiliated to Golestan University of Medical Sciences. Advances in Nursing & Midwifery 2018;27:32-6.
50. Haroutunian P, Alsabri M, Kerdiles FJ, Adel Ahmed Abdullah H, Bellou A. Analysis of Factors and Medical Errors Involved in Patient Complaints in a European Emergency Department. Advanced Journal of Emergency Medicine 2018;2:e4.
51. Hendrie J, Sammartino L, Silvapulle MJ, Braitberg G. Experience in adverse events detection in an emergency department: nature of events. Emergency Medicine Australasia 2007;19:9-15.
52. Hendrie J, Yeoh M, Richardson J, et al. Case-control study to investigate variables associated with incidents and adverse events in the emergency department. Emergency Medicine Australasia 2017;29:149-57.
53. Henneman PL, Blank FSJ, Smithline HA, et al. Voluntarily reported emergency department errors. Journal of patient safety 2005;1:126-32.
54. Hitti E, Tamim H, Bakhti R, Zebian D, Mufarrij A. Impact of Internally Developed Electronic Prescription on Prescribing Errors at Discharge from the Emergency Department. The Western Journal of Emergency Medicine 2017;18:943-50.
55. Hoang R, Sampsel K, Willmore A, Labre KY, Thiruganasambandamoorthy V, Calder LA. Remember that patient you saw last week: characteristics and frequency of patients experiencing anticipated and unanticipated death following ED discharge. CJEM Canadian Journal of Emergency Medical Care 2021;23:767-71.
56. Horwitz LI, Meredith T, Schuur JD, Shah NR, Kulkarni RG, Jenq GY. Dropping the baton: a qualitative analysis of failures during the transition from emergency department to inpatient care. Annals of emergency medicine 2009;53:701-10.e4.
57. Hussain F, Cooper A, Carson-Stevens A, et al. Diagnostic error in the emergency department: learning from national patient safety incident report analysis. BMC emergency medicine 2019;19:77.
58. Kachalia A, Gandhi TK, Puopolo AL, et al. Missed and delayed diagnoses in the emergency department: a study of closed malpractice claims from 4 liability insurers. Annals of emergency medicine 2007;49:196-205.
59. Kallberg AS, Goransson KE, Florin J, Ostergren J, Brixey JJ, Ehrenberg A. Contributing factors to errors in Swedish emergency departments. International emergency nursing 2015;23:156-61.
60. Kannampallil TG, Manning JD, Chestek DW, et al. Effect of number of open charts on intercepted wrong-patient medication orders in an emergency department. Journal of the american medical informatics association 2018;25:739-43.
61. Kelly JJ, Farley H, O'Cain C, et al. A survey of the use of time-out protocols in emergency medicine. Joint Commission Journal on Quality & Patient Safety 2011;37:285-8.
62. Kerr D, Klim S, Kelly AM, McCann T. Impact of a modified nursing handover model for improving nursing care and documentation in the emergency department: A pre- and post-implementation study. International Journal of Nursing Practice 2016;22:89-97.
63. Khaleghi P, Akbari H, Masoudi Alavi N, Motalebi Kashani M, Batooli Z. Identification and analysis of human errors in emergency department nurses using SHERPA method. International emergency nursing 2022;62:101159.
64. Kiechle ES, McKenna CM, Carter H, et al. Medication Allergy and Adverse Drug Reaction Documentation Discrepancies in an Urban, Academic Emergency Department. Journal of Medical Toxicology: Official Journal of the American College of Medical Toxicology 2018;14:272-7.
65. Kiymaz D, Koc Z. Identification of factors which affect the tendency towards and attitudes of emergency unit nurses to make medical errors. Journal of Clinical Nursing 2018;27:1160-9.
66. Klasco RS, Wolfe RE, Wong M, et al. Assessing the rates of error and adverse events in the ED. American journal of emergency medicine 2015;33:1786-9.
67. Kukielka E, Jones R. Medication Safety in the Emergency Department: A Study of Serious Medication Errors Reported by 101 Hospitals From 2011 to 2020. Patient Safety (2689-0143) 2022;4:49-59.
68. Kulstad EB, Sikka R, Sweis RT, Kelley KM, Rzechula KH. ED overcrowding is associated with an increased frequency of medication errors. American journal of emergency medicine 2010;28:304-9.
69. Kwok ESH, Clapham G, White S, Austin M, Calder LA. Development and implementation of a standardised emergency department intershift handover tool to improve physician communication. BMJ Open Quality 2020;9:02.
70. Lombardi D, Gaston-Kim J, Perlstein D, et al. Preventing wrong-patient electronic orders in the emergency department. Journal of Clinical Outcomes Management 2016;23(12):550-4.
71. Lu TC, Tsai CL, Lee CC, et al. Preventable deaths in patients admitted from emergency department. Emergency medicine journal 2006;23:452-5.
72. Macias Maroto M, Solis Carpintero L. [Medication administration errors at an emergency service: knowing to decrease risk]. Revista Espanola de Salud Publica 2018;92:28.
73. Maughan BC, Lei L, Cydulka RK. ED handoffs: observed practices and communication errors. American journal of emergency medicine 2011;29:502-11.
74. Mendes JR, Lopes M, Vancini-Campanharo CR, Okuno MFP, Batista REA. Types and frequency of errors in the preparation and administration of drugs. Einstein 2018;16:eAO4146.
75. Mitchell Scott B, Considine J, Botti M. Medication errors in ED: Do patient characteristics and the environment influence the nature and frequency of medication errors? Australasian Emergency Nursing Journal 2014;17:167-75.
76. Monfaredi S, Gaeeni M, Koohpaei A, Khandan M. Identification and assessment of nursing task errors in emergency department using SHERPA technique and offering remedial strategies. International emergency nursing 2021;59:101103.
77. Mostafa LS, Sabri NA, El-Anwar AM, Shaheen SM. Evaluation of pharmacist-led educational interventions to reduce medication errors in emergency hospitals: a new insight into patient care. Journal of Public Health 2020;42:169-74.
78. Murray KA, Belanger A, Devine LT, Lane A, Condren ME. Emergency department discharge prescription errors in an academic medical center. Baylor University Medical Center Proceedings 2017;30:143-6.
79. Nassief K, Azer M, Watts M, Tuala E, McLennan P, Curtis K. Emergency department care-related causal factors of in-patient deterioration. Australian Health Review 2022;46:35-41.
80. Negash G, Kebede Y, Hawaze S. Medication errors in the adult emergency unit of a tertiary care teaching hospital in Addis Ababa. Archives of Pharmacy Practice 2013;4(4):147-53.
81. Nunez S, Hexdall A, Aguirre-Jaime A. Unscheduled returns to the emergency department: an outcome of medical errors? Quality & Safety in Health Care 2006;15:102-8.
82. Ogilvie M, Nissen L, Kyle G, Hale A. An evaluation of a collaborative pharmacist prescribing model compared to the usual medical prescribing model in the emergency department. Research In Social & Administrative Pharmacy 2022;18:3744-50.
83. Okafor N, Mazzillo J, Miller S, et al. Improved Accuracy and Quality of Information During Emergency Department Care Transitions. The Western Journal of Emergency Medicine 2017;18:459-65.
84. Okafor N, Payne VL, Chathampally Y, Miller S, Doshi P, Singh H. Using voluntary reports from physicians to learn from diagnostic errors in emergency medicine. Emergency medicine journal 2016;33:245-52.
85. Owens K, Palmore M, Penoyer D, Viers P. The Effect of Implementing Bar-Code Medication Administration in an Emergency Department on Medication Administration Errors and Nursing Satisfaction. Journal of Emergency Nursing 2020;46:884-91.
86. Pahlevan D, Jandaghi J, Shaeeri M, Razavi MR, Abdollahpour A, Kermani A. Classification and assessment of medication errors in the emergency unit of a hospital in Iran by SHERPA. World Family Medicine 2018;16:107-12.
87. Patanwala AE, Sanders AB, Thomas MC, et al. A prospective, multicenter study of pharmacist activities resulting in medication error interception in the emergency department. Annals of emergency medicine 2012;59:369-73.
88. Patanwala AE, Warholak TL, Sanders AB, Erstad BL. A prospective observational study of medication errors in a tertiary care emergency department. Annals of emergency medicine 2010;55:522-6.
89. Payes Peich M, Juanes Borrego AM, Garcia Pelaez M, Marinosa Marre M, Iglesias Lepine ML, Altimiras Ruiz J. Medication errors detected in a hospital's emergency services. [Spanish]. Atencion Farmaceutica 2011;13(2):113-6.
90. Perez-Diez C, Real-Campana JM, Noya-Castro MC, Andres-Paricio F, Reyes Abad-Sazatornil M, Bienvenido Povar-Marco J. [Medication errors in a hospital emergency department: study of the current situation and critical points for improving patient safety]. Emergencias 2017;29:412-5.
91. Pham JC, Story JL, Hicks RW, et al. National study on the frequency, types, causes, and consequences of voluntarily reported emergency department medication errors. Journal of emergency medicine 2011;40:485-92.
92. Raban MZ, Walter SR, Pont LG, Cheung L, Strumpman D, Westbrook JI. The potential impact of an electronic medication management system on safety‐critical prescribing errors in an emergency department. Journal of Pharmacy Practice & Research 2019;49:108-15.
93. Riaz MK, Hashmi FK, Bukhari NI, Riaz M, Hussain K. Occurrence of medication errors and comparison of manual and computerized prescription systems in public sector hospitals in Lahore, Pakistan. PLoS ONE [Electronic Resource] 2014;9:e106080.
94. Rothschild JM, Churchill W, Erickson A, et al. Medication errors recovered by emergency department pharmacists. Annals of emergency medicine 2010;55:513-21.
95. Salmasian H, Blanchfield BB, Joyce K, et al. Association of Display of Patient Photographs in the Electronic Health Record With Wrong-Patient Order Entry Errors. JAMA network open 2020;3:e2019652.
96. Shitu Z, Aung MMT, Tuan Kamauzaman TH, Ab Rahman AF. Prevalence and characteristics of medication errors at an emergency department of a teaching hospital in Malaysia. BMC health services research 2020;20:56.
97. Sinopoulou V, Rutter P, Price G, Heald V, Kaba S, Kwok J. Implementing an emergency department pharmacy service and its effect on medication safety. International Journal of Pharmacy Practice 2021;29:394-6.
98. Sklar DP, Crandall CS, Loeliger E, Edmunds K, Paul I, Helitzer DL. Unanticipated death after discharge home from the emergency department. Annals of emergency medicine 2007;49:735-45.
99. Solano JJ, Dubosh NM, Anderson PD, Wolfe RE, Edlow JA, Grossman SA. Hospital ward transfer to intensive care unit as a quality marker in emergency medicine. American journal of emergency medicine 2017;35:753-6.
100. Stasiak P, Afilalo M, Castelino T, et al. Detection and correction of prescription errors by an emergency department pharmacy service. CJEM Canadian Journal of Emergency Medical Care 2014;16:193-206.
101. Su CF, Chu CM, Yuan YJ, et al. Use of a Modified Fishbone Diagram to Analyze Diagnostic Errors in Emergency Physicians. Journal of Acute Medicine 2017;7:149-57.
102. Tejedor Fernandez M, Montero-Perez FJ, Minarro Del Moral R, Gracia Garcia F, Roig Garcia JJ, Moyano AMG. Design and implementation of a patient safety program for a hospital emergency department: how to do it a patient safety culture. Emergencias 2013;25:218-27.
103. Thomas M, Mackway-Jones K. Incidence and causes of critical incidents in emergency departments: a comparison and root cause analysis. Emergency medicine journal 2008;25:346-50.
104. Thomas M, Morton R, Mackway-Jones K. Identifying and comparing risks in emergency medicine. Emergency medicine journal 2004;21:469-72.
105. Turner JS, Courtney RD, Sarmiento E, Ellender TJ. Frequency of safety net errors in the emergency department: Effect of patient handoffs. American journal of emergency medicine 2021;42:188-91.
106. Vaidotas M, Yokota PKO, Negrini NMM, et al. Medication errors in emergency departments: is electronic medical record an effective barrier? Einstein 2019;17:eGS4282.
107. Vazin A, Zamani Z, Hatam N. Frequency of medication errors in an emergency department of a large teaching hospital in southern Iran. Drug Healthcare & Patient Safety 2014;6:179-84.
108. Venkatesh AK, Curley D, Chang Y, Liu SW. Communication of Vital Signs at Emergency Department Handoff: Opportunities for Improvement. Annals of emergency medicine 2015;66:125-30.
109. Vergel FJM, Ricoma ND, Parra DM, Ortiz FR, Rodriguez AMC, Ruiz LL. Diagnostic agreement between a comprehensive, fast-track community hospital emergency department and the corresponding referral hospital. Emergencias 2010;22:282-5.
110. Watts H, Nasim MU, Sweis R, Sikka R, Kulstad E. Further characterization of the influence of crowding on medication errors. Journal of Emergencies Trauma & Shock 2013;6:264-70.
111. White AA, Wright SW, Blanco R, et al. Cause-and-effect analysis of risk management files to assess patient care in the emergency department. Academic emergency medicine 2004;11:1035-41.
112. Yamamoto LG. Reducing emergency department charting and ordering errors with a room number watermark on the electronic medical record display. Hawai'i Journal of Medicine & Public Health : A Journal of Asia Pacific Medicine & Public Health 2014;73:322-8.
113. Zargarzadeh AH, Mousavi S, Omranian R. Emergency department medication errors in a large teaching hospital in centre of Iran. International Research Journal of Pharmacy 2018;9(8):48-52.
114. Zeraatchi A, Talebian MT, Nejati A, Dashti-Khavidaki S. Frequency and types of the medication errors in an academic emergency department in Iran: The emergent need for clinical pharmacy services in emergency departments. Journal of Research in Pharmacy Practice 2013;2:118-22.
115. Zhang E, Hung SC, Wu CH, Chen LL, Tsai MT, Lee WH. Adverse event and error of unexpected life-threatening events within 24hours of ED admission. American journal of emergency medicine 2017;35:479-83.
116. مقدم مع, پاشا مق, نژاد كرمانی سق. حقوق انتشار این اثر، متعلق به دانشگاه علوم پزشکی و خدمات بهداشتی درمانی شهید بهشتی می باشد. 1 گزارش مختصر علل خطاهاي تشخيصي منجر به شکایت در بخش اورژانس: یك گزارش مختصر. Iranian Journal of Emergency Medicine 2019;6:96-101.

**Supplementary Appendix 1. Search strategy for the systematic review**

**CINAHL with Full Text via EBSCO**(1963+):

| S15 | S11 NOT S14 |
| --- | --- |
| S14 | S12 OR S13 |
| S13 | TI(review or metaanalysis or meta-analysis) |
| S12 | (MH "Child+" OR MH "Adolescence+") NOT (MH "Aged+") |
| S11 | S3 AND S6 AND S10 |
| S10 | S7 OR S8 OR S9 |
| S9 | TI(emergency N5 (error* or erroneous* or mistake* or near-miss* or close-call* or sentinel-event* or oversight or omission* or preventable-adverse-event*)) OR AB(emergency N5 (error* or erroneous* or mistake* or near-miss* or close-call* or sentinel-event* or oversight or omission* or preventable-adverse-event*)) |
| S8 | TI(error* N5 (communicat* or knowledge* or diagnos* or medication* or drug* or dose* or dosage* or procedur* or treatment* or allerg* or physical-exam* or test* or lab* or imaging or consult* or abnormal* or vital-signs or discharg* or document* or checklist* or prevent*)) OR AB(error* N5 (communicat* or knowledge* or diagnos* or medication* or drug* or dose* or dosage* or procedur* or treatment* or allerg* or physical-exam* or test* or lab* or imaging or consult* or abnormal* or vital-signs or discharg* or document* or checklist* or prevent*)) |
| S7 | TI(omission* or omit* or neglect* or overlook* or forget* or forgot* or (fail* N2 (act or detect* or diagnos* or document* or chart* or recogni* or identif* or report*)) or misdiagnos* or wrong-site* or wrong-patient* or wrong-drug* or wrong-dos* or wrong-medic* or wrong-procedur* or wrong-treatment* or incorrect-dos* or incorrect-site* or incorrect-patient* or incorrect-drug* or incorrect-medic* or incorrect-procedur* or incorrect-treatment* or misprescri* or mis-prescri* or inaction) OR AB(omission* or omit* or neglect* or overlook* or forget* or forgot* or (fail* N2 (act or detect* or diagnos* or document* or chart* or recogni* or identif* or report*)) or misdiagnos* or wrong-site* or wrong-patient* or wrong-drug* or wrong-dos* or wrong-medic* or wrong-procedur* or wrong-treatment* or incorrect-dos* or incorrect-site* or incorrect-patient* or incorrect-drug* or incorrect-medic* or incorrect-procedur* or incorrect-treatment* or misprescri* or mis-prescri* or inaction) |
| S6 | S4 or S5 |
| S5 | TI(error* or erroneous* or mistake* or near-miss* or close-call* or sentinel-event* or oversight or omission* or preventable-adverse-event*) OR AB(error* or erroneous* or mistake* or near-miss* or close-call* or sentinel-event* or oversight or omission* or preventable-adverse-event*) |
| S4 | (MH "Health Care Errors+") OR (MH "Sentinel Event") |
| S3 | S1 OR S2 |
| S2 | TI(emergency N2 (care or department* or room* or medicine or service* or unit* or ward* or physician* or nurs*)) OR AB(emergency N2 (care or department* or room* or medicine or service* or unit* or ward* or physician* or nurs*)) |
| S1 | (MH "Emergency Patients" OR MH "Physicians, Emergency" OR MH "Emergency Service+" OR MH "Emergency Medicine" OR MH "Emergency Nursing+" OR MH "Emergency Care+") |

**Cochrane Central Register of Controlled Trials (CCTR) via Ovid** (1991+):

| **#** | **Query** | **Results from 22 Nov 2022** |
| --- | --- | --- |
| 1 | (emergency adj2 (care or department* or room* or medicine or service* or unit* or ward* or physician* or nurs*)).ab,hw,ti. | 20,696 |
| 2 | (error* or erroneous* or mistake* or near-miss* or close-call* or sentinel-event* or oversight or omission* or preventable-adverse-event*).ab,hw,ti. | 25,858 |
| 3 | (omission* or omit* or neglect* or overlook* or forget* or forgot* or (fail* adj2 (act or detect* or diagnos* or document* or chart* or recogni* or identif* or report*)) or misdiagnos* or wrong-site* or wrong-patient* or wrong-drug* or wrong-dos* or wrong-medic* or wrong-procedur* or wrong-treatment* or incorrect-dos* or incorrect-site* or incorrect-patient* or incorrect-drug* or incorrect-medic* or incorrect-procedur* or incorrect-treatment* or misprescri* or mis-prescri* or inaction).ab,hw,ti. | 10,938 |
| 4 | (error* adj5 (communicat* or knowledge* or diagnos* or medication* or drug* or dose* or dosage* or procedur* or treatment* or allerg* or physical-exam* or test* or lab* or imaging or consult* or abnormal* or vital-signs or discharg* or document* or checklist* or prevent*)).ab,ti. | 2,838 |
| 5 | or/3-4 | 13,612 |
| 6 | (emergency adj5 (error* or erroneous* or mistake* or near-miss* or close-call* or sentinel-event* or oversight or omission* or preventable-adverse-event*)).ab,hw,ti. | 99 |
| 7 | or/5-6 | 13,658 |
| 8 | 1 and 2 and 7 | 245 |
| 9 | (neonat* or newborn* or infan* or toddler* or p?ediatric* or child* or girl* or boy* or adolesc* or teen* or youth).ti. | 147,475 |
| 10 | (Journal: Conference Proceeding or Conference Proceeding).pt. | 188,777 |
| 11 | (review or metaanalysis or meta-analysis).ti,pt. | 8,301 |
| 12 | or/9-11 | 331,218 |
| 13 | 8 not 12 | 153 |

**Embase via Ovid**(1974+):

| **#** | **Query** | **Results from 22 Nov 2022** |
| --- | --- | --- |
| 1 | exp Emergency Service, Hospital/ or Emergency Medicine/ or Emergency Nursing/ | 59,852 |
| 2 | (emergency adj2 (care or department* or room* or medicine or service* or unit* or ward* or physician* or nurs*)).ab,kf,ti,dq. | 289,895 |
| 3 | or/1-2 | 313,880 |
| 4 | error/ or omission error/ or medical errors/ or diagnostic errors/ or medication errors/ or near miss, healthcare/ | 286,856 |
| 5 | (error* or erroneous* or mistake* or near-miss* or close-call* or sentinel-event* or oversight or omission* or preventable-adverse-event*).ab,kf,ti,dq. | 559,188 |
| 6 | or/4-5 | 779,502 |
| 7 | omission error/ | 88 |
| 8 | (omission* or omit* or neglect* or overlook* or forget* or forgot* or (fail* adj2 (act or detect* or diagnos* or document* or chart* or recogni* or identif* or report*)) or misdiagnos* or wrong-site* or wrong-patient* or wrong-drug* or wrong-dos* or wrong-medic* or wrong-procedur* or wrong-treatment* or incorrect-dos* or incorrect-site* or incorrect-patient* or incorrect-drug* or incorrect-medic* or incorrect-procedur* or incorrect-treatment* or misprescri* or mis-prescri* or inaction).ab,kf,ti,dq. | 347,275 |
| 9 | (error* adj5 (communicat* or knowledge* or diagnos* or medication* or drug* or dose* or dosage* or procedur* or treatment* or allerg* or physical-exam* or test* or lab* or imaging or consult* or abnormal* or vital-signs or discharg* or document* or checklist* or prevent*)).ab,ti. | 61,316 |
| 10 | or/7-9 | 403,266 |
| 11 | 3 and 6 and 10 | 3,929 |
| 12 | (emergency adj5 (error* or erroneous* or mistake* or near-miss* or close-call* or sentinel-event* or oversight or omission* or preventable-adverse-event*)).ab,kf,ti,dq. | 979 |
| 13 | or/11-12 | 4,444 |
| 14 | exp juvenile/ not exp adult/ | 2,468,999 |
| 15 | limit 13 to (books or chapter or conference abstract) | 1,784 |
| 16 | (review or metaanalysis or meta-analysis).ti,pt. | 3,429,451 |
| 17 | or/14-16 | 5,739,218 |
| 18 | 13 not 17 | 2,000 |

**MEDLINE via Ovid**(1946+ and Epub Ahead of Print, In-Process & Other Non-Indexed Citations and Ovid MEDLINE(R) Daily):

| **#** | **Query** | **Results from 22 Nov 2022** |
| --- | --- | --- |
| 1 | exp Emergency Service, Hospital/ or Emergency Medicine/ or Emergency Nursing/ | 113,114 |
| 2 | (emergency adj2 (care or department* or room* or medicine or service* or unit* or ward* or physician* or nurs*)).ab,kf,ti. | 191,196 |
| 3 | or/1-2 | 231,662 |
| 4 | medical errors/ or diagnostic errors/ or medication errors/ or near miss, healthcare/ | 70,822 |
| 5 | (error* or erroneous* or mistake* or near-miss* or close-call* or sentinel-event* or oversight or omission* or preventable-adverse-event*).ab,kf,ti. | 427,601 |
| 6 | or/4-5 | 475,406 |
| 7 | (omission* or omit* or neglect* or overlook* or forget* or forgot* or (fail* adj2 (act or detect* or diagnos* or document* or chart* or recogni* or identif* or report*)) or misdiagnos* or wrong-site* or wrong-patient* or wrong-drug* or wrong-dos* or wrong-medic* or wrong-procedur* or wrong-treatment* or incorrect-dos* or incorrect-site* or incorrect-patient* or incorrect-drug* or incorrect-medic* or incorrect-procedur* or incorrect-treatment* or misprescri* or mis-prescri* or inaction).ab,kf,ti. | 261,449 |
| 8 | (error* adj5 (communicat* or knowledge* or diagnos* or medication* or drug* or dose* or dosage* or procedur* or treatment* or allerg* or physical-exam* or test* or lab* or imaging or consult* or abnormal* or vital-signs or discharg* or document* or checklist* or prevent*)).ab,ti. | 43,528 |
| 9 | or/7-8 | 301,915 |
| 10 | 3 and 6 and 9 | 2,044 |
| 11 | (emergency adj5 (error* or erroneous* or mistake* or near-miss* or close-call* or sentinel-event* or oversight or omission* or preventable-adverse-event*)).ab,kf,ti. | 670 |
| 12 | or/10-11 | 2,400 |
| 13 | (exp infant/ or exp child/ or adolescent/) not exp adult/ | 2,092,429 |
| 14 | (review or metaanalysis or meta-analysis).ti,pt. | 3,420,152 |
| 15 | or/13-14 | 5,311,080 |
| 16 | 12 not 15 | 1,825 |
| 17 | from 16 keep 1-1824 | 1,824 |

**Web of Science Core Collection via Clarivate Analytics**(Science Citation Index Expanded 1975+ & Emerging Sources Citation Index 2015+):

| #9 | #7 NOT #8 and Meeting Abstract (Exclude – Document Types) |
| --- | --- |
| #8 | (TI=(neonat* or newborn* or infan* or toddler* or p?ediatric* or child* or girl* or boy* or adolesc* or teen* or youth)) OR TI=(review or metaanalysis or meta-analysis) |
| #7 | #1 AND #2 AND #6 |
| #6 | #3 OR #4 OR #5 |
| #5 | TS=(emergency NEAR/5 (error* or erroneous* or mistake* or near-miss* or close-call* or sentinel-event* or oversight or omission* or preventable-adverse-event*)) |
| #4 | TS=(error* NEAR/5 (communicat* or knowledge* or diagnos* or medication* or drug* or dose* or dosage* or procedur* or treatment* or allerg* or physical-exam* or test* or lab* or imaging or consult* or abnormal* or vital-signs or discharg* or document* or checklist* or prevent*)) |
| #3 | ((TS=(omission* or omit* or neglect* or overlook* or forget* or forgot*)) OR TS=(fail* NEAR/2 (act or detect* or diagnos* or document* or chart* or recogni* or identif* or report*))) OR TS=(misdiagnos* or wrong-site* or wrong-patient* or wrong-drug* or wrong-dos* or wrong-medic* or wrong-procedur* or wrong-treatment* or incorrect-dos* or incorrect-site* or incorrect-patient* or incorrect-drug* or incorrect-medic* or incorrect-procedur* or incorrect-treatment* or misprescri* or mis-prescri* or inaction) |
| #2 | TS=(error* or erroneous* or mistake* or near-miss* or close-call* or sentinel-event* or oversight or omission* or preventable-adverse-event*) |
| #1 | emergency NEAR/2 (care or department* or room* or medicine or service* or unit* or ward* or physician* or nurs*) (Topic) |

**Supplementary Appendix 2. Free-text comments collected during Round 1 and 2 of the Delphi process**

Color coding:

Items to add/advantages

Items to remove/drawbacks

Other suggestions

**General comments on the proposed ED Safety Checklist**

| Whereas emergency room requires timely intervention, safety for self and patient is still paramount to prevent avoidance errors. Therefore, checklist is one of those tools that can help guide actions to ensure safe and adequate emergency care |
| --- |
| Unfortunately there is no certain check list in our ED ward. I think this would help ER physicians for better practice and management. |
| It's very useful. We are in the process of standardizing all day-to-day practices in the ED |
| Helps the residents in clinical decision making and improving patient safety, provided residents do not blindly check off the list. |
| Seems like checklists could have a big impact on patient safety in the ED. The non-linear flow of action in the ED will be one of the biggest challenges here. |
| Accurate checklists help us identify all the steps required for a process. We will be able to identify vital tasks and prioritize them. We will be able to divert your attention, time, and effort in completing these before others. |
| congratulations for the nice project which i believe will bring uniformity in ED across the global village and hence serve a lot of life |
| I hope there will be such an effective and generally accepted checklist in every ED department to help us for better management and to reduce certain errors in practice. |
| We are working hard to develop the most complete checklist possible to apply in the ED but fortunately we were able to participate in the survey of CERTAIN ED Delphi process and we found it to be very perfect and appropriate compatible with our emergency system. |
| This should be a template form for EHR and not a separate document or program. I think it is very well put together and thought through. |
| Excellent questionnaire with a number of thought provoking recommendations and a lot of emphasis on what makes really good ED care |
| checklist usage is very important and it will help in minimising the mistakes in a chaotic emergency department |
| Thank you so much for creating and suggesting such a comprehensive safety net - I am curious to learn about the weighting of these parameters by the participants. |
| Even though checklists, all needn't be done for every parent and sometimes some other tests will be done as per the physicians expertise. |
| Checklists may be sometimes pain in the neck especially in overcrowded ED with less medical personnel and/or MD |
| Unsure that I would use it. |
| I fear that this list will lead to the prescription of many tests, procedures, therapies (including antibiotics!)... |
| Too long. Should focus on initial and discharge and hand-off only |
| I worry about spending all your time ensuring checklist are checked and forgetting about the patient |
| I must be honest: I am not sure that a general checklist for all patients can be implemented. In busy EDs, physicians will not adhere to this for all patients. I would rather focus on specific situation at risk. But to be honnest again, I am not very confident that this can help and be used... |
| The checklist is too long with too many unspecific tasks. It does not address many of the risks that exist in the ED, e.g. risk of falls, risk of pressure ulcers, distended bladder. I also lack the question: Have risks of deterioration been identified and communicated to team? |
| This is a great academic exercise but I worry about the real world applicability given how incredibly long this is. Is there a president for anything like this in other areas of medicine that is in use? In general would try to focus on the questions/domains that have some reasonable evidence that they ever get missed - the highest yield areas - and stick to that. |
| A discussion of the process if using this checklist in a particular order that is most suitable should be tailored with the checklist itself for better orientation |
| If there were some groups for each ED, it would be better. For example my ED had more than 100 new emergency patients one day, it maybe different need from the basis community ED. FYI for all above-mentioned. |
| To the question above about where to embed - sure for med students would be great in a book, or even perhaps early stage residents, but for the rest - different sections of this I think have different value. For example a 're evaluation' checklist or 'pre disco' - SUPER helpful - as a pop up, the 'after initial eval' less so. |
| Remove the right hand column in every zone, just have a blank checkbox. Would be good to use for "safety Rounds" by ED manager as they round on bedside RN. Could make checkmark with dry erase marker on laminated card. |
| The emergency department safety checklist should be designed to swiftly identify and draw attention of the health care team to the most sick and confirm life saving interventions needed and instituted. Therefore at each stage of critical patient handling in the ED , the safety checklist should provide for documenting a particular life saving intervention made by the team eg correcting hypoglycemia, arresting hemorrhage, securing airway or proper patient positioning at triage or at any other station of patient care where any of these might be necessary. Therefore the last sentence on each of the first 1 pages of the checklist could be " urgent life saving interventions done...................to ensure that the teams do not move the patient to the next stage of care before instituting the urgently needed measures within their means |
| The treatment plan and the medication to be administered must be evaluated or discussed with a limited number of doctors, since in some cases it can lead to different treatments being suggested and not one by unanimous decision. |
| The checklist is very good and comprehensive. Adding schemes or mnemonics will make is more practical and memorable |
| Should admission be a separate category? Potentially could be considered either in the handoff or discharge section and will overlap some with both, but really it seems like a distinct entity. |
| Consider a provider/physician checklist and a RN checklist. |
| Add a checklist for preventing and controlling infections. |
| All items on checklists are important elements of good clinical work but almost all of them are already an integral part of the services provided. If the staff is required to use checklist for obvious things like doing an H&P and order labs, this could result in significantly increased load of work and the "click fatigue" that is already a huge problem in health care. |
| We do not have Physician assistant, aiming to have still not yet available |
| We currently have procedures and policies and have most of this in place but the checklist can help other hospitals. This can help the residents and medical students think through the cases. |
| Include diagnostic reasoning schemes / diagnostic errors safeguards |
| Patient preferences and goals of care is redundant if the plan of care is implemented I don't think that something like working disposition should be on checklist |
| Look very well prepared Cultural/ social and religious preferences need to be acknowledged and documented |

**Triage section**

| We use ESI(emergency severity index) for triage patient at first 🡪 consider requirement of emergency medical intervention (intubated, unresponsive, pulselessness, apneic, severe  respiratory distress, profound hypotension or hypoglycemia), high risk of deterioration, acutely altered mental status, severe pain, altered vital signs (HR, RR, SpO2; BP and Temperature not explicitly included) |
| --- |
| If the safety checklist is to be Universal and for use in any department then the triage section should include - - Assess for bleeding -Blood sugar -Temperature -In surgical, Obstetrics and Gynecology emergency departments omission of assessing for bleeding in a patient at triage (especially those with concealed hemorrhage ) means deterioration of the patient. -Hypoglycemia in the critically ill if missed at triage leads to poor treatment outcome; Hyperglycemia too complicates patient manage if not diagnosed swiftly at triage. - Temperature shouldn't be missed among the vitals at triage |
| Identity, level of conciousness and vital signs are crucial initial steps in the ED mgt of any patient Whereas pain is a common presentation, it can be addressed only after the above , but within the ED |
| We check BS by glucometer in patient with decrease in level of consciousness. we also check tempreture in triage |
| Depending on the kind of patient, sometimes it won't be easy to take the patient's identity like in the case of trauma or critically ill patients |
| It is also important to ask or measure the patient's temperature. |
| Identity may be checked after making sure the patient is not red or orange triage |
| I would include the pregnancy state or puerperium and allergies. |
| Presentation symptom should be added to the triage list. |
| Assessing the main reason for ED visit is important |
| Australia uses the Australasian Triage Scale which mandates minimum data to be collected during triage assessment (The triage assessment involves a combination of the presenting problem and general appearance of the patient, and may be combined with pertinent physiological observations) |
| Most important asspect of the cheklist is the identity chek, since other data is routinelly entered in the triage form, and identity is often assumed based on what is entered in the computer system by a clerk. |
| Vital signs should be split into individual items on the checklist based on each vital sign (e.g. one item for BP, one item for SpO0 etc), as they are often not checked simultaneously |
| The presence of these elements is essential, but so is the interpretation grid, to avoid under-evaluation and over-evaluation of the priority |
| We combined the assessment of the vital signs with a scoring system. we called the early warning scoring system. Check this document <https://www.rcplondon.ac.uk/projects/outputs/national-early-warning-score-news-0>. Comprise RR, SpO2, temperature, sBP, HR, consciousness. |
| Adding "Assess Temperature" in the triage phase can be useful to complete the vitals assessment, especially whether infectious risk should be considered |
| All these information are necessary to avoid severe errors and to collocate the patient in the right path |
| Is there any data supporting that we get the identity wrong at the point of triage? This is not a failure mode I have seen and in general with checklists would remove items that don't represent any fallibility |
| Pt identity is already performed in all patients |
| One doesn't die from pain itself and pain is subjective |
| Presence and level of pain should probably be split. Presence is likely to be important, especially depending on the location. I am not sure level is important. |
| Important to ask RNs as they are end user here. Also why is temperature not listed in vital signs? In my opinion this is the most frequently missed vital sign and should be checked at triage. |
| The purpose of triage is to identify patients needing immediate resuscitation; to assign patients to a predesignated patient care area, thereby prioritizing their care; and to initiate diagnostic/therapeutic measures as appropriate. |
| A section of the summary of just presenting complaint and brief history might help to be included just after the vitals section at initial encounter |
| Assessing the demographics, vitals, mental status and pain score are essential in a triage area and omitting one of them can lead to under triage or over triage |
| Determine need for immediate eval ie ESI or triggers for emergency intervention |
| Revaluate pain response to therapy |
| Also evaluate work of breathing and circulation (such as Capillary refill, Blood pressure, central vs peripheral pulse, heart rate, filling situation)... |
| Temperature can be included in the "evaluate vital signs" section, rather than being a stand alone in the checklist |
| Finger stick glucose just in case we have a neurological deterioration or known insuline user |
| Comorbidities, medications and allergies should be recorded if the patient is to wait before entering the ED |
| Point number two where there is temperature we can generally write vital signs |
| Identify primary family contact for the patient and way to keep in touch with him if needed  Ask for allergies |
| ECG for those who are over 40 years |
| Finger-stick glucose should be perform generally but not all. In some situation, it's not reasonable to do finger-stick glucose, eg young patient with no prior abnormal history, come to ED causes of mild cutting wound of hand, normal consciousness. Doing this test in this circumstance can cause more pain, incurred cost, complaint of patient and more work for medical staff |
| Need for urgent ECG/ cardiovascular/ neurological intervention |

**Diagnostic evaluation section**

| Once the vitals and mental state are evaluated, the rest of the items like pain, allergies, as well as other tests can always follow once the life-threatening emergencies are addressed. Including them in the initial checklist, would make it unnecessarily long and time consuming, for an ED setting. These can probably be included in a "next actions" page after the initial contact page... during full clerkship |
| --- |
| It is difficult to make definite decisions on plan of care on the initial few minutes of patient assessment always. Ater initial assessment and stabilisation, perhaps it can be done |
| When it comes to documentation in the ED, patient care is the priority then documentation later so initially you will do basic documentation then later after stabilizing your patient you will do full documentation discussion with the ED team or other departments most of the time will be done later after the initial investigation and management unless in direct cases |
| Where is 'focused history including key components of presenting complaint and risk factors for conquering items on ddx' or something like that? In my ED shift yesterday it struck me in our high acuity area that all of these items we're often quite good at - but the history is often forgotten or overlooked by residents and represents significant risk of diagnostic error |
| It is also important to ask or measure the patient's height and weight during this stage. This is helpful for the administration of certain drugs. |
| If the aim is to be a general checklist then the ones I marked agree with are important to include, but if in the future in EMR then maybe best to tailor list to the differential diagnosis so as to avoid checklist fatigue |
| Suggest a question about potential risks during ED stay, e.g. risk of falls, risks of delirium, risk of self harm. Nursing aspects are missing. |
| Many of these factors lead to the systematic prescription of examinations and procedures (e.g., iv line, which is very often unnecessary and responsible for a longer stay in the emergency room). In our department, we consider that triage indicates the place of installation/consultation box, and that examinations and procedures must be prescribed and performed in the box, not upstream, because this is a frequent source of overconsumption of diagnostic and therapeutic procedures, and of delay in the care of patients in the triage sector (this time indicator is essential for us). Triage team (nurse) prescribes radiology examinations for extremity trauma without severity. This saves a lot of time in the patient circuit |
| I would not place ordering of exam in a checklist... I try to "disagree" with several items so the checklist can be shorter. If not, it is unlikely that there will be close adherence |
| Discuss the goals of care (for all patients ) and code status (if relevant) Shall we include the indication of each interventions in the explanation / rationale |
| Evaluate risk of fall, risk of pressure ulcers and patient autonomy |
| What about individual items for patient's history has been reported home therapy list has been reported Main patient's complaint has been focused? I think it would be useful to check items of actions that are often at risk of being overlooked in the overcrowded ED. Reporting all the available actions which can be done can make the check list too long. Instead of "Consider if urinary catheter is needed" an item "Consider if urinary output should be monitored and how?" can encourage noninvasive monitoring when it is possible |
| Istat - many places don't have consider cultures - be careful planting seeds that can lead to over testing complete all notes - many people don't work in environments where that is reasonable or feasible |
| Whether of not to perfom POCUS or order x-rays is part of the medical workup. Better if the checklist focuses on things that are easily forgotten/omitted. |
| I think all this happens in our heads while we are seeing a patient.Going through a checklist might help less experienced providers (students, residents, NP/PA with less experience) |
| 0. Goals of care and resuscitation are not relevant in most ED patients and therefore cannot be the standard in caring for each patient given time constraints. Clearly if a patient presents with life threatening disease this may need to be part of the conversation 0. Patients do not survive based on documentation Ideally a running online chart with brief history, care plan and pending tests should be part of the record as soon as one takes leave of the patient |
| Same comment about pain as the triage comment. The ED care team seems a little problematic here. It is probably important, but it sort of depends on who that refers to (which isn't really clear here). |
| Is initial assessment done by the bedside RN? I am assuming yes. If provider should make that clear. And then I would say the first half of these questions should be aimed nursing. I am answering the first half (until urinary catheter) s if these are done by RN, the second half should be done by provider. My answers apply as if provider were asking them. |
| Initial Assessments process used to identify and treat life-threatening problems, Assessment concentrating on Level of Consciousness, Cervical Spinal Stabilization, Airway, Breathing, and Circulation. |
| What is up for discussion will end up being just marked without being done  Maybe say that you should discuss with the consultant about the further plan, considering that with us, Younger doctors have to present every patient to the senior (specialist, consultant) in the service |
| There is a fundamental question in the answers here related to what you’re trying to focus on. What juice is worth the squeeze. A pilots checklist doesn’t include ‘sit down in the seat’ - so what items (like ‘review history and presenting complaint’) are in that category? For every additional item on the checklist, it’s less likely to get used. |
| The assessment, investigative and diagnostic process is patient-centered, timely, reliable, and of high-quality. A working diagnosis or list of possible diagnoses will be developed and discussed with the patient. Treatment and assessment may occur concurrently. Repeated assessment may be required. |
| It is impossible to do a diagnostic evaluation without checking or considering most of these things. A checklist should include critical steps that tend to be overlooked. |
| Discuss with nurse where to have the patient waiting and how to perform diagnostic tests (in bed, in chair, in waiting room) |
| All these are crucial inclusions, and not time consuming. They've to be part of the care for any patient |
| I prefer to separate pregnancy from lactation because the pregnancy can change the approach and the plan significantly. however, lactation is only important when prescribing medications. |
| ask for covid, influenza immunization status. ask if the patient has had covid episodes |
| Review the type of patient diet or if the patient should be NPO |
| If this is after primary survey and stabilization, pregnancy and breastfeeding should have been recorded then patient preferences are in the part where we discuss plan of care |
| Have to check on personal habits like smoking if that is needed in this study as pollution and not smoking should be the cause of the results. Around industry areas , so may be location can be looked into. |
| At this stage, talking to the patient's primary physician (if any) can be considered to get to know any pertinent medical/surgical history and/or current health concerns being managed just in case patient might have forgotten some information that could be critical to their care at that moment. |

**Treatment prescription section**

| Most of the listed items are important in general patient care, but in the ED, the urgent potentially life-threatening issues are to be addressed. The rest can be addressed even outside the ED. In some settings, and for most drugs, general adult/ paed doses can be used for emergencies, rather than strictly weighing everyone at initial encounter |
| --- |
| We don't routinely weigh adults in our ED, at best we estimate weight |
| Although i find this form to have all the date i find it to large for a routin use esp for trivial mediction (paracetamol iv etc) Consideration for ventilator/fludis/analgesia is in my opinion for the first cheklist NPO order should be included in triage assesment and the first encounter) MAybe consider including all the current therapy reviewed item ? |
| We must define "other contraindications" Antibiotic use must be considered only for sepsis patients. |
| Using a scheme to arrange the items will help the memorization. Can we include the 1 S mnemonic: 0. Site of care (ICU. HDU, Ward) 0. Supportive treatment (e.g. Fluids, Oxygen) 0. Symptomatic treatment (e.g Ananelgasic, anti-emetic, etc) 1. specific treatment (antibiotics, thrombolysis for MI etc ) 1. speciality referral or input. |
| Adding specific therapies can make the checklist much longer. MAybe only therapies at risk of being forgotten can be specifyed? E.g. home therapy or analgesic therapy or fluids in case of a patient with NPO? Add item: have therapies to be repeated been scheduled? (e.g.ATB?) Consider if oxygen/ventilatory support therapy is needed could be inserted in the previous step when vitals and all immediate patients requirements are evaluated |
| Better to make the list short and with things that are easily forgotten, e.g. lactating |
| Oxygen - would be interesting to know if this is EVER missed. Checklists, like surveys, need to be as short as possible. with all additional questions you lose uptake. This one I think is low yield (unless any evidence it gets missed?) Pt weight - such a small number of meds/fluids when you need this. Low yield and frankly not feasible in most ED situations where it's even relevant. |
| Regular meds can be ordered later if they are staying for long |
| Most medications are not weight based in the ED, although a weight may be necessary, more likely than not it will need in an adult population and cannot be mandated due to time constraints. Regular medication administration is a by product, for the most part, of ED crowding. If patients are expected to have long stays in the ED or stay goes beyond the industry standard, say 0 hours, then a review of which of the patients regular medications are needed during ED stay takes on greater meeting |
| Regarding the last item, if a procedure is urgent/emergent it should be done regardless of PO status. If not, it probably isn't that important here. I can't think of a time I have ever seen a procedure delayed in the ED due to PO status. |
| Assuming this is for provider |
| Taking your medicine as prescribed or medication adherence is important for controlling chronic conditions, treating temporary conditions, and overall long-term health and well-being. A personal connection with your health-care provider or pharmacist is an important part of medication adherence |
| For high risk meds, ensure correct route and dose and renal adjust. |
| Simplify - put special considerations  (pregnancy, contraindications, etc.), for analgosedation special flow chart  with us, patients do not stay in the ED for so long that they need to order a meal (very rarely) |
| Some items on list are pretty obvious, you generally don't need a checklist to remember to decide if a patient needs oxygen or ventilatory support. Checklists should be to help you remember to consider steps that tend to get overlooked. |
| The last two here are too general to be actionable |
| Patients are monitored following the administration of medications |
| Wt is rarely relevant in treating adults |
| Accurate weight estimation is often difficult in ED |
| Estimate patient weight if not known by the patient or patient unconscious) also check the ideal body weight for further treatment |
| Having "prescribe all the relevant..." will extend the list without significant help |
| The patient may be reevaluated with regard to disease-specific therapies by relevant clinics during working hours |
| Ensure patient comprehension and understanding of the medication dosage, route and side-effects/ interactions |

**Treatment administration section**

| This is generally important in any drug administration |
| --- |
| I would add another variable: Check if the drug is a high-risk med that needs double verification (electrolytes, insulin, LASA (looks alike, sound alike, etc.) |
| Maybe this should use the 1 rights of medication administration or the 7 rights, aim to align to standard that is used widely in nursing medication administration à 7 rights of administration: individual, medication, dose, time, route, documentation, response |
| I find that the safety system for drug administration should be more robust than trying to check if the drug has already been administered... or if some other procedure has already been made... |
| I would advise against having the same items twice for prescription and administration |
| Some duplication from prior page - would suggest streamlining this. |
| This is for RN |
| 2x2 eye principles in drug administration  Check again the vital signs before giving the drug (e.g. is there still a hypertension before giving a antihypertensive therapy) |
| The nurses should also check for the intravenous access. |
| I do not consider the detection of all vital signs before administering the drug to be of little importance (unless it is foreseen for the specific administration), however, I believe an effective "quick look" to verify the need to detect them is essential |
| asses, change, re-assess |
| Reviewing vitals can't be included for before drug administration. This can be reserved for specific meds in specific conditions. E.g BP/glucose lowering drugs, one is compelled to check these first. Otherwise for the other drugs, it's unnecessary. The routine ED vitals monitoring protocol would suffice |
| Pain score |
| Explain to the patient briefly before administrating certain drugs with side effects. |
| If the vital signs worsen, the primary physician should be notified immediately. |
| Additionally, we should provide pain score |
| consider any pre-treatmnet that needs to be administered |

**Invasive procedures section**

| I think resuscitation equipment has to always be there |
| --- |
| Ideally, each procedure needs a separate checklist |
| I would add another variable: Check if all the material and instrumental its complete before beginning the procedure |
| We don't routinely get written consent for procedures performed in our ED. Maybe we should? |
| Check this document https://rcem.ac.uk/wp-content/uploads/0000/00/Invasive_Procedure_Checklist_for_EDs_May0006.pdf |
| Add presence of all needed material and check that is functioning, availability of additional necessary devices, plan B for unpredicted complications |
| What about adding item: check full availability of the procedural material? |
| I think a general procedure checklist is too unspecific to be useful. |
| We use WHO checklist modified for ED procedures |
| Informed consent is understood in regard to most ED procedures particularly if not performing them is life threatening. I would caution not to waste signifcant time on consent when a patient is unstable |
| Unclear what test might mean here - test often means diagnostic. I think this should just be for procedures. |
| When sending blood samples in particular, GXM, always ensure to ask 0 identifiers open endedly. when performing procedure, ensure correct site. |
| No need to verify allergies and team member roles.  Patients' blood type should be confirmed. |
| Reassess and record vital signs at the end of the procedure |
| I am worried about creating a list that its so extensive that it will not be used |
| consider Retained guidewire risk |
| The checklists for procedures have to be specific and generic lists should not be used Any procedural errors or complications need to be documented |

**Patient reassessment section**

| By time revelation comes, patient condition and status, should be clear |
| --- |
| With my stay in the ED, I think this is one of the very important parts of emergency medicine RE-EVALUATION |
| This would be good if built into EMR that it is timed so as to meet decision target timeframes |
| For non-priority patients in the emergency room, is there a set routine for monitoring vital signs? Same for priority patients How do we define priorities, on triage and on location? |
| What about adding Assess persistence or resolution of patient's main complaints? |
| Alert the nurses for any urgent orders. |
| This is also too vague to be useful. |
| I would call out with vital signs - and most recent set of vitals. different than trend. I would also call out 'review any pending, unresolved labs or imaging' |
| Please see my prior comments on documentation and pain |
| Last item seems kind of vague. Not really sure what it means. |
| Probably less value in checklist approach to patient re-eval than for other instances. |
| Clinical re-assessment of the patient incl. vital signs and GCS and body check (difference to the first assessment) |
| These ones all represent easily missed essentials - that seems like the sweet spot here. |
| As the emergency management is preferentially focused on the rapid stabilization of the patient and transfer to the respective units for the definitive workup and management. Reviewing differential diagnosis may fall out of favor as a necessary tool in the ED. |
| For boarded patients, a treatment plan not only drugs prescription are suggested |
| Update patient and family after first diagnostic workup results are available |
| Consider need of additional tests and treatments based on diff. dg. and patient clinical condition and treatment response |
| Highlight significant investigation findings that warrant closer monitoring eg elevated serum potassium  Highlight key clinical examination findings that require particular monitoring eg signs of drug toxicity for instance tendon reflexes for those on MgSO4 |
| Discuss plan of care with the specialty involved Discourage unnecessary boarding and ensure expeditious and safe disposition Monitor LOS and wait time (not a clinical indicator but the impact is significant) |

**Handoff section**

| Horrible party of ED, if not well considered it leads to a lot of unnecessary delays, mismanagement and sometimes losing life |
| --- |
| Documentation is needed. |
| According to the system I-PASS, a short code of the illness severity (Stable, "Watcher," Unstable) and synthesis by the receiver would be helpful. |
| This is a significant safety check point |
| Can we use the Ipass the baton checklist here? https://www.profsolutions.com/webres/File/physicians/sample_forms_library/MD%00Connection%00Newsletter%00INSERT%001_01%00x%001_1_FNL.pdf |
| What about a specific item after patient identification and location: Discuss patient's main complaint/reason for ED visit |
| I wouldn't re-create the wheel on this one. IPASS exists - consider using that. I think the 'watcher' or acuity conversation and contingency planning is actually really helpful in IPASS |
| A discussion of results can be electronic, as in a check list a summary sentence of patients care and plan on a dashboard, as it is in our institution, rarely needing verbal clarification |
| Should also add assessment of stability/patient condition. Maybe IV access as well. |
| Handouts can be vague, consider to add a reminder to verbalize the assessment, what you think is going and and what have you excluded. |
| Discuss the will of the patient in dependence to the test results. |
| These are all fine - but I would encourage you to not boil the ocean here - don’t bite off pass off - instead link to already validated tools like iPass. |
| We should build a structured clinical handover at all points of transition of care, including when a patient leaves or returns to the ED, and whenever staﬀ changeovers occur. |
| I am convinced that also the hand-off among the nursing staff would benefit from this checklist (which even if the contents could be different ), would provide a frame of mind for an effective hand-off . |
| All of this should be in an electronic format with info readily viewable to oncoming or admitting physicians or nurse. Direct communication is needed only if critical actions or information needs to be transmitted or there is info which cannot easily be placed on an electronic dashboard |
| Do a joint doctor-nurse recap after handover with rapid visual patients’ re-assessment |
| Checking for synthesis by the receiver, might not necessarily apply. The other issues are enough, especially that most of the initial management plan is well documented, and the ED might have several patients anyway. I would rather emphasize proper documentation to sort this, rather than wasting valuable time to assess synthesis by the receiver |
| Discuss any system or health facility or Departmental challenges or gaps affecting patient care and how to get them addressed in the shift starting |
| Discuss any non-clinical events related to the patient Brief about errors that may have happened in patient management and ensure detailed documentation by the outgoing team of such events Detailed brief regarding difficult patient or family |

**Discharge section**

| I think a doctor has to think of the worst-case scenario that will happen to a patient once discharged before doing so. we can consider that in the checklist I sometimes see a lot of readmissions and lost life due to poor decisions during discharging laboratory and radiological results should not be the standard of discharging patients. sometimes they are all normal but the pt is sick, with my experience patient hx and physical examinations has to be the golden standard for discharging patients. patient vital signs have to be reviewed during discharge. consider patient's chief complaint while discharging think of masking IV ant pain which they won't use at home |
| --- |
| Talk about all the warning signs for the patient's specific condition that need a new medical consultation in the ED. |
| Arranging an appointment for a needed outpatient clinic should be considered. |
| Provide also writing information to the patient and the family. Make sure that the GP get the same information and plan further follow up with the GP |
| Need of health education intervention and of GP/ambulatory follow up |
| Check if there are devices still to be removed (PVC, urinary catheter, etc.) |
| This is the weakest checklist. Some additions: 'review any pending, unresolved labs or imaging' Also a 'cognitive time out' - review diagnostic information and differential diagnosis. Determine if anything missing/doesn't fit together. Consider alternatives. Etc. |
| Not sure what "medications and test orders" means. Based on the rationale, I assume medications means prescriptions, but so it should probably be called that. Typically we don't order any tests out of the ED. I also don't know what a "discharge note" is or means. Does this mean just completing and signing the visit note? Or is this supposed to refer to something else? Either way, probably doesn't need to be done immediately at the time of discharge. |
| What does discharge note mean? EHR note? then yes. but call it encounter or visit note. |
| For Geri pts, ensure Next of kin to accompany patient home. |
| To check patients identity every time you visit the room will in the end be awkward, should absolutely be done on first contact and then we all need to remember to recheck if you are managing too many patients at the same time and might be getting them mixed up. |
| Discuss whether help and support is needed at home (care at home) |
| Will need more definition around what is ‘review clinical condition’ what does that mean? The discuss ones are great and very needed |
| We should add medication safety in this checklist |
| If we patient is at deposition time differential diagnosis must have already been reviewed and a determination that pt is safe or unsafe for d/c then made  Nursing is responsible for removing equipment ie IV lines and should communicate abnormal vital signs at disposition time to the md |
| Explain also logistic details of follow-up program (possible to have a dedicated administrative figure for this) |
| Provide patient's with writing checklist with alarm symptoms, medication , follow up, etc... |
| Perfect. For those discharged home, could probably need a contact from the ED, who can be called in case of any questions. So we could add ,"check that an emergency contact has been provided at discharge" |
| Discuss the follow up plan with the patient if needed. |
| I Must say that I like the discharge protocol. It now covers everything |
| In case that invasive devices can not be removed, eg: Foley catheter, discuss with patient/ family the reason, how to care and abnormal signs for re-examination. Patient can be safe to discharge but we can can not confirm 100%, we need to explain risks or complications that patient may face to and when patient need to come back for check. |
| Encourage patient /family to let the department know what they thing should be done to improve quality of patient care |
| Also discuss plans in case an emergency/ explain red flags |
